# Decision aids that facilitate elements of shared decision making in chronic illnesses: a systematic review

**DOI:** 10.1186/s13643-019-1034-4

**Published:** 2019-05-20

**Authors:** Thomas H. Wieringa, Rene Rodriguez-Gutierrez, Gabriela Spencer-Bonilla, Maartje de Wit, Oscar J. Ponce, Manuel F. Sanchez-Herrera, Nataly R. Espinoza, Yaara Zisman-Ilani, Marleen Kunneman, Linda J. Schoonmade, Victor M. Montori, Frank J. Snoek

**Affiliations:** 1 Department of Medical Psychology, Amsterdam Public Health Research Institute, Amsterdam UMC, Vrije Universiteit Amsterdam, De Boelelaan 1117, Amsterdam, the Netherlands; 2 Knowledge and Evaluation Research Unit, Mayo Clinic, Rochester, MN USA; 3 Division of Endocrinology, Department of Internal Medicine, “Dr. Jose E. González” University Hospital, Autonomous University of Nuevo Leon, Monterrey, Nuevo Leon Mexico; 4 Plataforma INVEST Medicina UANL-KER Unit Mayo Clinic, KER Unit México, “Dr. Jose E. González” University Hospital, Autonomous University of Nuevo Leon, Monterrey, Nuevo Leon Mexico; 5 Department of Medicine, Stanford University School of Medicine, Stanford, CA USA; 6 College of Public Health, Temple University, Philadelphia, PA USA; 7 Medical Decision Making, Department of Biomedical Data Sciences, Leiden University Medical Center, Leiden, the Netherlands; 8 Medical Library, VU University, Amsterdam, the Netherlands

**Keywords:** Chronic illnesses, Decision aids, Shared decision making

## Abstract

**Background:**

Shared decision making (SDM) is a patient-centered approach in which clinicians and patients work together to find and choose the best course of action for each patient’s particular situation. Six SDM key elements can be identified: situation diagnosis, choice awareness, option clarification, discussion of harms and benefits, deliberation of patient preferences, and making the decision. The International Patient Decision Aid Standards (IPDAS) require that a decision aid (DA) support these key elements. Yet, the extent to which DAs support these six key SDM elements and how this relates to their impact remain unknown.

**Methods:**

We searched bibliographic databases (from inception until November 2017), reference lists of included studies, trial registries, and experts for randomized controlled trials of DAs in patients with cardiovascular, or chronic respiratory conditions or diabetes. Reviewers worked in duplicate and independently selected studies for inclusion, extracted trial, and DA characteristics, and evaluated the quality of each trial.

**Results:**

DAs most commonly clarified options (20 of 20; 100%) and discussed their harms and benefits (18 of 20; 90%; unclear in two DAs); all six elements were clearly supported in 4 DAs (20%). We found no association between the presence of these elements and SDM outcomes.

**Conclusions:**

DAs for selected chronic conditions are mostly designed to transfer information about options and their harms and benefits. The extent to which their support of SDM key elements relates to their impact on SDM outcomes could not be ascertained.

**Systematic review registration:**

PROSPERO registration number: CRD42016050320.

**Electronic supplementary material:**

The online version of this article (10.1186/s13643-019-1034-4) contains supplementary material, which is available to authorized users.

## Background

Shared decision making (SDM) is a patient-centered approach in which clinicians and patients work together to find and choose (by taking into account the best available evidence, as well as the patients’ problems, values, preferences, and contexts) the best course of action for each patient’s particular situation [1], an approach that is pertinent to the care of patients with chronic conditions [2]. Decisions in the context of self-management of chronic conditions differ from one-time decisions, as in the former decisions can often be reconsidered [2]. Six key elements of SDM can be identified from the literature: situation diagnosis, choice awareness, option clarification, discussion of harms and benefits, patient preferences deliberation, and making the decision [1–4]. As noted by Stiggelbout and others [5, 6], SDM promotes actions that are needed, wanted, and more likely to be implemented. A shared understanding and treatment focused on patients’ health and life goals, as well as a stronger clinician-patient relationship, may also be facilitated by SDM [7, 8].

An SDM interaction starts with a diagnostic conversation (situation diagnosis) [1]. This opening first focuses on understanding the patient’s situation and establishing the aspects that require action [1, 4]. When multiple reasonable options are available, then the clinician should indicate this and highlight the importance of the patient’s preferences in deciding on the course of action (choice awareness) [3]. Subsequently, the patient and clinician deliberate about the way each option fits and accommodates within each patient’s situation (option clarification, discussion of harms and benefits, and patient preferences deliberation). Finally, a decision is made by the clinician and patient (making the decision) [2, 4].

SDM can be facilitated by decision aids (DAs) that have been developed for use by clinicians and patients, either during or in preparation for the clinical encounter [9–11]. DAs can help patients choose an option that is congruent with their values, reduce the proportion of patients remaining undecided and/or who play a passive role in the decision-making process, and improve patient knowledge, decisional conflict, and patient-clinician communication [11–15]. The International Patient Decision Aid Standards (IPDAS) Collaboration aims to enhance the quality and effectiveness of DAs by establishing an evidence-informed framework for improving their content, development, implementation, and evaluation [16]. The IPDAS Collaboration defines a DA as “a tool designed to help people participate in decision making about health care options” [9], and developed a minimal set of standards for qualifying a tool as a DA [17]. According to this minimal set, all SDM key elements, except making the decision, should be handled by a tool in order to regard it as a DA [17]. Despite this minimal set of qualifying criteria, investigators have found that fostering choice awareness through the use of a DA was not a prerequisite for fostering choice awareness per se during the encounter [18]. Therefore, it is unclear whether tools should support all qualifying IPDAS criteria for these tools to support SDM. Therefore, we define a DA in the current review as “any tool designed to support SDM.”

To the best of our knowledge, there is no empirical data to tell us which of the six key elements are supported by DAs and whether there is an association between support for these key elements and SDM outcomes. We hypothesize that DAs that cover multiple elements of SDM are more likely to have positive effects on SDM outcomes, as well as on patient-reported outcomes (PROs). With regard to surrogate and clinical outcomes, there is no reason to expect a consistent response. A previous systematic review of the effects of DAs found that more detailed DAs better improve knowledge and reduce some aspects of decisional conflict compared to simple DAs, and concluded that more research is needed to evaluate the level of detail needed in DAs [19]. The current review aims to meet this need by studying the SDM elements incorporated in DAs and their effect on SDM outcomes.

This review aims to (1) describe the SDM elements present in DAs for patients with common chronic conditions (cardiovascular, chronic respiratory diseases or diabetes) tested in randomized controlled trials (RCTs), and (2) determine an association between the key elements present and the effects of these DAs compared to usual care or active controls on SDM outcomes (e.g., conversation duration, patient participation, knowledge, and decisional conflict).

## Methods

The protocol of this systematic review was previously published [20] and registered in the International Prospective Register of Systematic Reviews (PROSPERO registration number: CRD42016050320; http://www.crd.york.ac.uk/PROSPERO). The review is reported according to the Preferred Reporting Items for Systematic Reviews and Meta-Analyses (PRISMA) guidelines [21]. Additional file 1 provides the PRISMA checklist.

### Study eligibility

We searched RCTs comparing the use of DAs (any tool designed to support SDM) to usual care or active controls (except other DAs) in adults with cardiovascular disease, chronic respiratory disease, or diabetes and measuring their impact on SDM and health outcomes (patient-reported, surrogate, and clinical outcomes). As described in detail previously [20], we selected chronic conditions that are most prevalent according to the World Health Organization [22–24], most likely to require self-management, and for which decisions may be revisited. We included all pertinent publications of an eligible study. There were no exclusions based on language or year of publication.

### Information sources and search strategy

To identify all relevant publications, we performed systematic searches, in collaboration with a medical librarian (LJS) in the bibliographic databases PubMed, Embase.com, Web of Science, CINAHL (through EBSCO), PsycINFO (through EBSCO), and the Cochrane Library from inception to November 7th, 2017. Search terms included MesH in PubMed, EMtree in Embase.com, Cinahl headings in Cinahl, indexed terms from the Thesaurus in PsycINFO, and free text terms. We used free text only in the Cochrane Library and Web of Science. Search terms compressing “shared decision making” were used in combination with “cardiovascular diseases” OR “chronic respiratory diseases” OR “diabetes.” Search results were limited to RCTs. Duplicate articles were excluded. All languages were accepted. The full search strategies for all databases can be found in Additional file 2. In early 2017, THW contacted by e-mail and queried SDM experts participating in the Facebook group “Shared@ Shared Decision Making Network,” and in the LinkedIn groups “Platform SDM GB” and “Shared Decision Making in Netherlands” for additional eligible studies. THW also reviewed trial registries including http://isrctn.com, http://narcis.nl, http://trialregister.nl, and http://www.clinicaltrials.gov. MFSH reviewed the reference lists from included studies.

### Study selection process

After deduplication, pairs of reviewers (two hired persons, GS-B, RRG, and THW), working independently and in duplicate, assessed each abstract for eligibility. Studies considered potentially eligible by at least one reviewer were included for the full-text phase. THW and RRG reviewed selected full-text articles independently and in duplicate. Disagreements were resolved by a third reviewer (GS-B or OJP).

### Data collection process

Data about study and DA characteristics, study quality, and outcomes were extracted by pairs of reviewers working in duplicate (two hired persons, RRG, MFSH, YZI, and THW) with conflict resolved by a third reviewer (GS-B, NRE, YZI, and RRG; YZI and RRG resolved conflicts of parts for which they did not collect data). We used the definitions in Table 1 to determine which key SDM components were present. Sets of three articles were used to train and calibrate reviewers through extraction and discussion of results among reviewers. Outcomes collected were those most proximate to the encounter of interest.

**Table 1:** Definitions for the key elements of SDM in decision aids (DAs)

Key element of SDM	Definitions for this study [4, 18]
Situation diagnosis	The DA explicitly describes the patient’s problem.
Choice awareness	The DA explicitly acknowledges that the patient’s situation is mutable, that there is more than one sensible way to address or change this situation, and that patient input matters in deciding how to proceed.
Option clarification	The DA explicitly lists and describes the options available.
Harms and benefits discussion	The DA explicitly explains the harms and benefits of the available options.
Patient preferences deliberation	The DA explicitly elicits the patient’s preferences or explicitly motivates the parties to discuss them.
Making the decision	The DA explicitly elicits the patient’s wish to make or defer a decision, asks for the patient’s choice, or describes the patient’s choice.

### Risk of bias in individual studies

OJP and THW independently assessed the risk of bias on outcome level at all domains of the Cochrane Collaboration’s tool for RCTs [25, 26], with disagreement resolved by consensus. Because blinding of patients and clinicians to the use of conversation aids is not possible, we ignored the two blinding factors. Otherwise, when one or more of the five other domains was regarded as being at high risk of bias, then the summary assessment of the risk of bias was “high.” If one or more domain was “unclear” and all others were “low risk,” then we summarized the risk of bias as “unclear.” If all domains were “low risk,” then the summary assessment of the risk of bias was “low.”

### Outcomes and data synthesis

Data on both SDM (e.g., conversation duration, patient participation, knowledge, and decisional conflict) and health outcomes (patient-reported, surrogate, and clinical outcomes) were collected. Standardized mean differences (SMDs) together with their 95% confidence intervals (95%-CIs) were calculated for continuous outcomes using Review Manager 5.3 [27]. Odds ratios (ORs) together with their 95% CIs were directly extracted from the reports. If the mean difference and/or its standard error (SE) and 95% CI were not presented in the article, then the SMD together with its 95% CI were calculated by entering the mean score/value per arm together with their standard deviations (SDs). If the 95% CI for an OR was not presented, then numbers for every cell in the 2 × 2 table were inserted into Review Manager 5.3 to be calculated. The SMD could not be calculated when only interquartile ranges were reported. We also summarize the data narratively according to our protocol [20].

### Missing data and author contact

All corresponding authors (or other authors if no response after approximately 6 weeks) of included studies were contacted through e-mail and, if no response, again approximately 4 weeks later (although originally planned, we did not contact authors by phone) to request missing data or clarifications. If the authors did not respond or could not provide a missing standard deviation needed to calculate the SMD, then the SD of the most comparable study with the same outcome and measurement instrument was imputed.

## Results

**Fig. 1:**
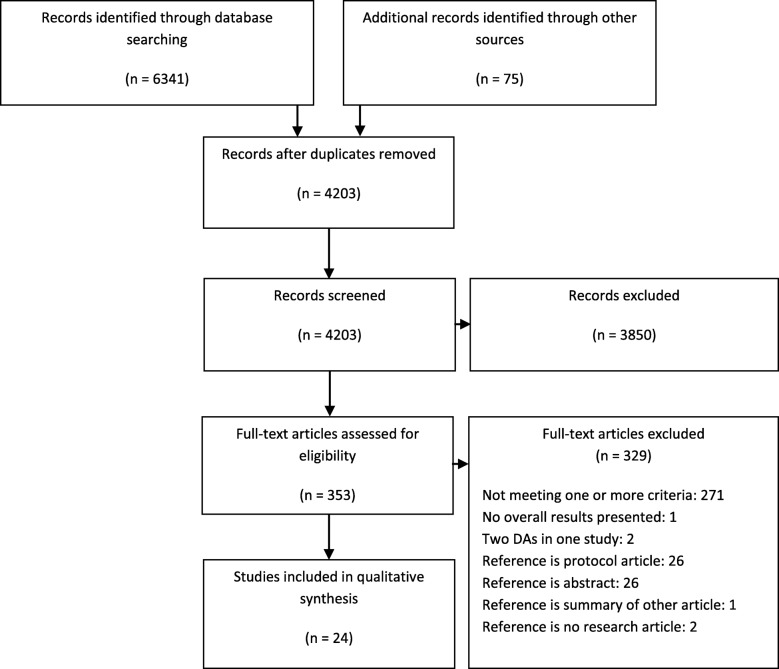
Flowchart of study selection

Figure 1 describes the flow of the study selection. Chance-adjusted inter-reviewer agreement (*k*) for eligibility was only fair (*k* = 0.3–0.4) [28]. We found 24 articles reporting on 23 RCTs of 20 DAs (10 DAs for cardiovascular disease, 2 DAs for respiratory diseases, and 8 DAs for diabetes). The effectiveness of Statin Choice was studied in three RCTs described in four articles meeting our criteria and The Diabetes Medication Choice Decision Aid was studied in two RCTs described in two separate articles. Other DAs were studied in one RCT described in one article. Additional file 3 presents the risk of bias assessment on the outcome level per study. Besides the study of Gagné et al. [29], all studies have an unclear or high risk of bias for all outcomes assessed in this review.

Table 2 shows the SDM elements supported per DA. The elements were described as “unclear” if the DAs were neither described clearly nor available for our inspection, and/or if reviewers were uncertain whether the regarding element was included in the DA. The option clarification element (included in 20 of 20 DAs; 100%) and the harms and benefits discussion (included in 18 of 20 DAs; 90%; unclear in two DAs) are the elements most commonly clearly included in the DAs. The other elements are less common and more uncertainty is present whether these elements are included, especially with regard to choice awareness (uncertain in 14 out of 20 DAs; 70%). All elements were clearly supported in four DAs (20%). Table 2 also shows the DA effects on SDM outcomes. We could not glean any association between SDM elements present in the DAs and SDM outcomes. Additional file 4 reports details of the DAs included here and Additional file 5 their impact on SDM and health outcomes. We imputed the SD for the decisional conflict outcome for Mann et al. [30] using the SD found by Weymiller et al. [31] for the same outcome in the same context.

**Table 2:** SDM elements included in DAs and DA effects on SDM outcomes

DA study, year	SDM elements in DA						SDM outcomes				
	Situation diagnosis	Choice awareness	Option clarification	Harms and benefits	Patient preferences	Making decision	Knowledge	Patient participation	Decisional conflict	Satisfaction	Time
Cardiovascular diseases											
Knops et al. 2014 [32]	✔	✔	✔	✔	✔	✔	↔	•	↔	↔	•
Man-Son-Hing et al. 1999 [33]	?	?	✔	✔	✔	✔	•	↔	↔	↔	•
Fraenkel et al. 2012 [34]	✔	?	✔	✔	✔	•	•	•	•	•	•
Thomas et al. 2013 [35]	✔	?	✔	✔	?	?	↔	•	↔	•	•
El-Jawahri et al. 2016 [36]	✔	✔	✔	?	?	?	↑	•	•	•	•
Korteland et al. 2017 [37]	✔	✔	✔	✔	✔	✔	•	•	↔	•	•
Thomson et al. 2007 [38]	?	?	✔	✔	✔	✔	↔	•	↑	•	•
Morgan et al. 2000 [39]	✔	?	✔	✔	?	?	↑	•	•	↔	•
Coylewright et al. 2016 [40]	•	?	✔	✔	✔	✔	•	↔	↔	•	•
McAlister et al. 2005 [41]	✔	✔	✔	✔	✔	✔	•	•	↑	•	•
Respiratory diseases											
Gagné et al. 2017 [29]	✔	?	✔	✔	✔	✔	↔	•	↔	•	•
Slok et al. 2016 [42]	✔	?	✔	?	✔	✔	•	•	•	•	•
Diabetes											
Huang et al. 2017 [43]	✔	✔	✔	✔	✔	•	•	•	↔	•	•
Statin Choice [30, 31, 44, 45]	✔	?	✔	✔	•	✔	↑	↑	↑/↔	↑	↔
Mathers et al. 2012 [46]	?	?	✔	✔	✔	?	•	•	↑	•	↔
Heisler et al. 2014 [47]	✔	?	✔	✔	✔	✔	↔	•	↔	•	•
Bailey et al. 2016 [48]	✔	?	✔	✔	✔	✔	↑	•	↑	•	•
Denig et al. 2014 [49]	✔	✔	✔	✔	✔	✔	•	•	•	•	•
Diabetes Medication Choice [50, 51]	•	?	✔	✔	✔	•	↔	↑	↔	•	•
den Ouden et al. 2017 [52]	•	?	✔	✔	✔	✔	•	•	•	•	•

Elements: • = not present; ? = unclear; ✔ = present; Outcomes: • = not reported; ↔ = no statistically significant effect; ↑ = favored DA

## Discussion

This review presents an overview of chronic care DAs developed and tested in RCTs, SDM elements they support, and their effects on SDM outcomes and health outcomes. Most DAs support the clarification of options and the discussion of their benefits and harms, while other elements are less prevalent. Almost all trials were at an unclear or high risk of bias, and no association between SDM elements supported in the DA on the one hand and SDM outcomes achieved versus control on the other hand could be determined.

### SDM elements handled by DAs

Our analysis of SDM elements supported is consistent with previous literature stating that most DAs focus and are tested on providing information or discussing choices rather than on creating empathic conversations [53]. We could not, however, estimate the relationship between the extent to which DAs support SDM elements and SDM outcomes.

Possibly, some SDM elements may have been left out of DAs by design. This choice may depend on what features were thought most important by the developers (e.g., patient education, risk communication, preference elicitation, or patient empowerment). The importance of incorporation of SDM elements in DAs may be situation-dependent, but the way this works is unclear. Future research should clarify this situation-dependence and eventually inform possible reconsideration of the IPDAS minimum standards for DA qualification [17].

### DA effects

The inability to find any empiric association between features present and SDM outcomes prevents us from using this evidence base to make recommendations about the content of DAs for use in patients with chronic conditions. Multiple factors potentially explain the varying effects, including the following: whether a patient decision aid or conversation aid is used [10], chronicity of conditions [2], design process [54, 55], context, target population [19], and degree of detail needed [19]. Future studies may assess the dependency of DA effects on these factors and their interactions with the SDM elements.

### Difficulties faced

Some difficulties were faced when conducting this review. A major difficulty during the article selection was the suboptimal reporting of DA characteristics. The aim of DAs is not always explicitly described and if described, it still may be questionable whether implementing SDM is implicitly aimed for as the concept of SDM itself is highly debatable [56]. Namely, a review found 31 separate concepts to explicate SDM [57]. Our ability to categorize whether SDM elements were present was limited by the fact that some DAs were not available and/or the description of the DA’s content was not clear and detailed. The latter is in line with the literature [58, 59]. Even when DAs were available and/or content was clearly described, it may not always be clear-cut whether or not an element is handled. Therefore, data regarding the SDM elements is based on reviewers’ judgments. Furthermore, it may sometimes be unclear whether or not a condition is chronic (e.g., aneurysms). These conditions were included in this review in order to be as comprehensive as possible, but the decisions to be made may not be reversible over time or only to a limited extent. These aspects may have resulted in the fair inter-rater agreement. Another difficulty was found in the large methodological heterogeneity across studies (e.g., measurement instruments, timing of outcome measurements, and presentation of results).

More guidance is needed on the reporting of SDM elements and DA aims, the measurement instruments to use in RCTs studying DA effects, as well as the timing of outcome measurements and the way results are presented in articles. Furthermore, the quality of RCTs studying DA effects can be improved. The new Standards for UNiversal reporting of patient Decision Aid Evaluation studies (SUNDAE) checklist seems to meet this need as it helps to ensure the high-quality reporting of DA evaluation studies, as well as its intelligibility and transparency [59].

### Strengths and limitations

This review is the first to report on SDM elements included in DAs developed for chronic conditions, and its relations to a range of SDM outcomes. This review underscores the importance of methodological improvement of DA evaluation studies, which hopefully will be attained by the new SUNDAE checklist [59].

Our review has some limitations. Since we were interested in the efficacy of DAs, we have limited our search strategy to RCTs [60], which may have led to exclusion of (well designed and developed) DAs that have not been tested in trials. Finally, we limited our search strategy to the most prevalent cardiovascular diseases, chronic respiratory diseases, and diabetes [22–24], an incomplete list of chronic diseases. This while a silver bullet of the literature probably brings to light what is happening in other chronic illnesses.

### Future research

Future research should focus on empirically testing which SDM elements should be included in DAs, and take situation-dependency into account. This warrants studies with a sound methodology and low risk of bias that are currently lacking.

## Conclusions

Tools to promote SDM for patients with chronic conditions support only some key recommended SDM elements thought to be important for SDM. The literature has not examined the relationship between explicit support for these elements in DAs and SDM outcomes.

## Additional files

Additional file 1: PRISMA checklist. (DOC 63 kb)

Additional file 2: Search histories bibliographic databases. (DOCX 25 kb)

Additional file 3: Risk of bias assessment. (DOCX 46 kb)

Additional file 4: DA information. (DOCX 77 kb)

Additional file 5: DA effects. (DOCX 64 kb)

## Abbreviations

95% CI: Confidence interval
DA: Decision aid
IPDAS: International Patient Decision Aid Standards
OR: Odds ratios
PRISMA: Preferred Reporting Items for Systematic Reviews and Meta-Analyses
PRO: Patient-reported outcomes
RCT: Randomized controlled trials
SD: Standard deviation
SDM: Shared decision making
SE: Standard error
SMD: Standardized mean difference
SUNDAE: Standards for UNiversal reporting of patient Decision Aid Evaluation studies
