# Supplementary material for: Decision aids that facilitate elements of shared decision making in chronic illnesses: a systematic review
Source: Syst Rev. 2019 May 20;8:121. doi: 10.1186/s13643-019-1034-4 (PMC6528254; doi:10.1186/s13643-019-1034-4)
Supplement: Supplementary file 2 — Search histories bibliographic databases. (DOCX 25 kb) [file 13643_2019_1034_MOESM2_ESM.docx]

# Additional file 2 search histories bibliographic databases

Table of Contents

[PubMed History November 7, 2017 2](#_Toc517427791)

[Embase.com History November 7, 2017 3](#_Toc517427792)

[Cinahl (Ebsco) History November 7, 2017 4](#_Toc517427793)

[PsycINFO (Ebsco) History November 7, 2017 6](#_Toc517427794)

[Web of Science History November 7, 2017 8](#_Toc517427795)

[The Cochrane Library (Wiley) History November 7, 2017 9](#_Toc517427796)

## PubMed History November 7, 2017(read from bottom up)

| Search | Query | Items found |
| --- | --- | --- |
| #6 | #1 AND #2 AND #3 AND #4 AND #5 | 1431 |
| #5 | (randomized controlled trial[pt] OR controlled clinical trial[pt] OR randomized controlled trials[mh] OR random allocation[mh] OR double-blind method[mh] OR single-blind method[mh] OR clinical trial[pt] OR clinical trials[mh] OR clinical trial*[tw] OR ((singl*[tw] OR doubl*[tw] OR trebl*[tw] OR tripl*[tw]) AND (mask*[tw] OR blind*[tw])) OR rct[tiab] OR intervention*[tiab] OR "latin square"[tw] OR placebos[mh] OR placebo*[tw] OR random*[tw] OR research design[mh] OR comparative study[pt] OR evaluation studies[pt] OR follow-up studies[mh] OR prospective studies[mh] OR cross-over studies[mh] OR control[tw] OR controll*[tw] OR prospectiv*[tw] OR volunteer*[tw]) NOT (animals[mh] NOT humans[mh]) | 6369008 |
| #4 | "Asthma"[Mesh] OR "Pulmonary Disease, Chronic Obstructive"[Mesh] OR asthma*[tiab] OR copd[tiab] OR chronic respiratory disease*[tiab] OR chronic obstructed pulmonary disease*[tiab] OR chronic obstructive airway disease*[tiab] OR chronic obstructive lung disease*[tiab] OR chronic bronchitis[tiab] OR emphysema[tiab] OR coad[tiab] OR chronic airflow obstruction*[tiab] OR "Diabetes Mellitus"[Mesh] OR diabetes[tiab] OR diabetic*[tiab] OR dm2[tiab] OR niddm[tiab] OR dm 2[tiab] OR t2d*[tiab] OR dm type 2[tiab] OR dm type II[tiab] OR dm1[tiab] OR iddm[tiab] OR dm 1[tiab] OR t1d*[tiab] OR dm type 1[tiab] OR dm type I[tiab] OR "Cardiovascular Diseases"[Mesh] OR cardiovascular disease*[tiab] OR cardiovascular disorder*[tiab] OR cardiovascular disturbance*[tiab] OR cardiovascular lesion*[tiab] OR cardiovascular syndrome*[tiab] OR cvd[tiab] OR myocardial ischem*[tiab] OR myocardial infarct*[tiab] OR heart disease*[tiab] OR coronary disease*[tiab] OR artery disease*[tiab] OR arterial disease*[tiab] OR heart attack*[tiab] OR heart failure*[tiab] OR cardiac failure*[tiab] OR high blood pressure*[tiab] OR hypertensi*[tiab] OR heart patient*[tiab] OR cerebrovascular disease*[tiab] OR cerebrovascular disorder*[tiab] OR vein thrombos*[tiab] OR embolism*[tiab] OR stroke*[tiab] OR cerebrovascular accident*[tiab] OR cva[tiab] OR cvas[tiab] OR vascular accident*[tiab] OR apoplexy[tiab] OR brain infarction*[tiab] | 3094052 |
| #3 | "Decision Support Techniques"[Mesh] OR "Decision Making, Computer-Assisted"[Mesh] OR tool*[tiab] OR aid[tiab] OR aids[tiab] OR intervention*[tiab] OR support*[tiab] OR instrument*[tiab] | 2975407 |
| #2 | "Patient-Centered Care"[Mesh] OR "Patient Participation"[Mesh] OR "Patient Preference"[Mesh] OR share*[tiab] OR sharing[tiab] OR engage*[tiab] OR patient centered*[tiab] OR patient centred[tiab] OR patient focused[tiab] OR sdm[tiab] OR prefer*[tiab] | 682674 |
| #1 | "Decision Making"[Mesh] OR "Clinical Decision-Making"[Mesh] OR Decision*[tiab] | 417397 |

##

## Embase.com History November 7, 2017(read from bottom up)

| Search | Query | Items found |
| --- | --- | --- |
| #6 | #1 AND #2 AND #3 AND #4 AND #5 | 2393 |
| #5 | 'intervention study'/exp OR 'clinical trial'/exp OR 'study design'/exp OR 'Latin square design'/exp OR 'comparative study'/exp OR 'controlled study'/exp OR 'crossover procedure'/exp OR 'double blind procedure'/exp OR 'randomized controlled trial'/exp OR 'single blind procedure'/exp OR 'prospective study'/exp OR 'comparative study'/exp OR 'evaluation study'/exp OR 'crossover procedure'/exp OR intervention*:ab,ti OR rct:ab,ti OR random*:ab,ti OR factorial*:ab,ti OR crossover*:ab,ti OR ‘cross over*’:ab,ti OR placebo*:ab,ti OR comparative:ab,ti OR comparing:ab,ti OR ‘evaluation stud*’:ab,ti OR trial*:ab,ti OR control*:ab,ti OR prospective:ab,ti OR assign*:ab,ti OR allocat*:ab,ti OR volunteer*:ab,ti OR ((singl*:ab,ti OR doubl*:ab,ti OR trebl*:ab,ti OR tripl*:ab,ti) AND (mask*:ab,ti OR blind*:ab,ti)) NOT ('animal'/exp NOT 'human'/exp) | 9043215 |
| #4 | 'asthma'/exp OR 'chronic obstructive lung disease'/exp OR asthma*:ab,ti OR copd:ab,ti OR ‘chronic respiratory disease*’:ab,ti OR ‘chronic obstructed pulmonary disease*’:ab,ti OR ‘chronic obstructive airway disease*’:ab,ti OR ’chronic obstructive lung disease*’:ab,ti OR ‘chronic bronchitis’:ab,ti OR emphysema:ab,ti OR coad:ab,ti OR ‘chronic airflow obstruction*’:ab,ti OR 'diabetes mellitus'/exp OR diabetes:ab,ti OR diabetic*:ab,ti OR dm2:ab,ti OR niddm:ab,ti OR ‘dm 2’:ab,ti OR t2d*:ab,ti OR ‘dm type 2’:ab,ti OR ‘dm type II’:ab,ti OR dm1:ab,ti OR iddm:ab,ti OR ‘dm 1’:ab,ti OR t1d*:ab,ti OR ‘dm type 1’:ab,ti OR ‘dm type I’:ab,ti OR 'cardiovascular disease'/exp OR ‘cardiovascular disease*’:ab,ti OR ‘cardiovascular disorder*’:ab,ti OR ‘cardiovascular disturbance*’:ab,ti OR ‘cardiovascular lesion*’:ab,ti OR ‘cardiovascular syndrome*’:ab,ti OR cvd:ab,ti OR ‘myocardial ischem*’:ab,ti OR ‘myocardial infarct*’:ab,ti OR ‘heart disease*’:ab,ti OR ‘coronary disease*’:ab,ti OR ‘artery disease*’:ab,ti OR ‘arterial disease*’:ab,ti OR ‘heart attack*’:ab,ti OR ‘heart failure*’:ab,ti OR ‘cardiac failure*’:ab,ti OR ‘high blood pressure*’:ab,ti OR hypertensi*:ab,ti OR ‘heart patient*’:ab,ti OR ‘cerebrovascular disease*’:ab,ti OR ‘cerebrovascular disorder*’:ab,ti OR ‘vein thrombos*’:ab,ti OR embolism*:ab,ti OR stroke*:ab,ti OR ‘cerebrovascular accident*’:ab,ti OR cva:ab,ti OR cvas:ab,ti OR ‘vascular accident*’:ab,ti OR apoplexy:ab,ti OR ‘brain infarction*’:ab,ti | 4845596 |
| #3 | 'decision support system'/exp OR tool*:ab,ti OR aid:ab,ti ORaids:ab,ti OR intervention*:ab,ti OR support*:ab,ti OR instrument*:ab,ti | 3580389 |
| #2 | 'patient participation'/exp OR 'patient preference'/exp OR share*:ab,ti OR sharing:ab,ti OR ‘patient centered*’:ab,ti OR ‘patient centred’:ab,ti OR ‘patient focused’:ab,ti OR sdm:ab,ti OR prefer*:ab,ti | 804965 |
| #1 | 'decision making'/exp OR 'clinical decision making'/exp OR decision*:ab,ti | 555692 |

## Cinahl (Ebsco) History November 7, 2017 (read from bottom up)

| Search | Query | Items found |
| --- | --- | --- |
| S6 | S1 AND S2 AND S3 AND S4 AND S5 | 435 |
| S5 | MH ("Clinical Trials+" OR "Quantitative Studies" OR "Study Design+" OR "Random Assignment" OR "Evaluation Research" OR "Comparative Studies") OR (PT Clinical trial) OR (TX clini* N1 trial*) OR (TX ((singl* N1 blind*) OR (singl* N1 mask*)) OR TX ((doubl* N1 blind*) OR (doubl* N1 mask*)) OR TX ((tripl* N1 blind*) OR (tripl* N1 mask*))) OR (TX randomi* control*) OR ((TX random* allocat*) OR (TX allocat* random*)) OR (TX placebo*) OR (TX (waitlist* OR (wait* AND list*)) AND (control* OR group))) OR ((TX "treatment as usual") OR (TX tau)) OR (TX (control* N3 (trial* OR study OR studies OR group*))) OR TX (rct OR intervention* OR “latin square” OR prospectiv* OR volunteer OR follow or factorial* OR crossover* OR “cross over” OR comparative OR comparing OR evaluation stud*) | 1552284 |
| S4 | MH (Asthma+ OR Pulmonary Disease, Chronic Obstructive+ OR Cardiovascular Diseases+ OR Diabetes Mellitus+ OR Diabetes Mellitus, Type 2 OR Diabetes Mellitus, Type 1+ OR Diabetes Mellitus, Gestational) OR TI (asthma* OR copd OR chronic respiratory disease* OR chronic obstructed pulmonary disease* OR chronic obstructive airway disease* OR chronic obstructive lung disease* OR chronic bronchitis OR emphysema OR coad OR chronic airflow obstruction* OR diabetes OR diabetic* OR dm2 OR niddm OR dm 2 OR t2d* OR dm type 2 OR dm type II OR dm1 OR iddm OR dm 1 OR t1d* OR dm type 1 OR dm type I OR cardiovascular disease* OR cardiovascular disorder* OR cardiovascular disturbance* OR cardiovascular lesion* OR cardiovascular syndrome* OR cvd OR myocardial ischem* OR myocardial infarct* OR heart disease* OR coronary disease* OR artery disease* OR arterial disease* OR heart attack* OR heart failure* OR cardiac failure* OR high blood pressure* OR hypertensi* OR heart patient* OR cerebrovascular disease* OR cerebrovascular disorder* OR vein thrombos* OR embolism* OR stroke* OR cerebrovascular accident* OR cva OR cvas OR vascular accident* OR apoplexy OR brain infarction*) OR AB (asthma* OR copd OR chronic respiratory disease* OR chronic obstructed pulmonary disease* OR chronic obstructive airway disease* OR chronic obstructive lung disease* OR chronic bronchitis OR emphysema OR coad OR chronic airflow obstruction* OR diabetes OR diabetic* OR dm2 OR niddm OR dm 2 OR t2d* OR dm type 2 OR dm type II OR dm1 OR iddm OR dm 1 OR t1d* OR dm type 1 OR dm type I OR cardiovascular disease* OR cardiovascular disorder* OR cardiovascular disturbance* OR cardiovascular lesion* OR cardiovascular syndrome* OR cvd OR myocardial ischem* OR myocardial infarct* OR heart disease* OR coronary disease* OR artery disease* OR arterial disease* OR heart attack* OR heart failure* OR cardiac failure* OR high blood pressure* OR hypertensi* OR heart patient* OR cerebrovascular disease* OR cerebrovascular disorder* OR vein thrombos* OR embolism* OR stroke* OR cerebrovascular accident* OR cva OR cvas OR vascular accident* OR apoplexy OR brain infarction*) | 448604 |
| S3 | MH ("Decision Support Techniques+" OR "Decision Support Systems, Clinical" OR "Decision Making, Computer Assisted") OR TI (tool* OR aid OR aids OR intervention* OR support* OR instrument*) OR AB (tool* OR aid OR aids OR intervention* OR support* OR instrument*) | 512902 |
| S2 | MH ("Patient Centered Care" OR "Consumer Participation") OR TI (share* OR sharing OR patient centered* OR patient centred* OR patient focused OR sdm OR prefer*) OR AB (share* OR sharing OR patient centered* OR patient centred* OR patient focused OR sdm OR prefer*) | 106376 |
| S1 | MH ("Decision Making" OR "Decision Making, Clinical" OR "Decision Making, Patient") OR TI decision* OR AB decision* | 106188 |

## PsycINFO (Ebsco) History November 7, 2017(read from bottom up)

| Search | Query | Items found |
| --- | --- | --- |
| S6 | S1 AND S2 AND S3 AND S4 AND S5 | 173 |
| S5 | DE "Treatment Effectiveness Evaluation" OR DE "Clinical Trials" OR DE "Placebo" OR TI (placebo* OR randomly) OR AB (placebo* OR randomly) OR TX randomi* OR TI trial OR AB trial OR TX ((singl* OR doubl* OR trebl* OR tripl*) N3 (blind* OR mask* OR dummy)) OR TI (control* N3 (trial* OR study OR studies OR group*)) OR AB (control* N3 (trial* OR study OR studies OR group*)) OR TI factorial* OR AB factorial* OR TI allocat* OR AB allocat* OR TI assign* OR AB assign* OR TI volunteer* OR AB volunteer* OR TI (crossover* OR cross over*) OR AB (crossover* OR cross over*) OR TX (quasi N5 (experimental OR random*)) OR AB (intervention* OR rct OR comparative OR comparing OR evaluation stud* OR prospective) OR TI (intervention* OR rct OR comparative OR comparing OR evaluation stud* OR prospective) | 835166 |
| S4 | DE ("Asthma" OR "Chronic Obstructive Pulmonary Disease" OR "Bronchial Disorders" OR "Pulmonary Emphysema" OR "Cardiovascular Disorders" OR "Aneurysms" OR "Arteriosclerosis" OR "Blood Pressure Disorders" OR "Cerebrovascular Disorders" OR "Embolisms" OR "Heart Disorders" OR "Hemorrhage" OR "Hypertension" OR "Ischemia" OR "Thromboses" OR "Diabetes" OR "Diabetes Insipidus" OR "Diabetes Mellitus" OR "Type 2 Diabetes" OR "Gestational Diabetes" OR DE "Blood Sugar") OR TI (asthma* OR copd OR chronic respiratory disease* OR chronic obstructed pulmonary disease* OR chronic obstructive airway disease* OR chronic obstructive lung disease* OR chronic bronchitis OR emphysema OR coad OR chronic airflow obstruction* OR diabetes OR diabetic* OR dm2 OR niddm OR dm 2 OR t2d* OR dm type 2 OR dm type II OR dm1 OR iddm OR dm 1 OR t1d* OR dm type 1 OR dm type I OR cardiovascular disease* OR cardiovascular disorder* OR cardiovascular disturbance* OR cardiovascular lesion* OR cardiovascular syndrome* OR cvd OR myocardial ischem* OR myocardial infarct* OR heart disease* OR coronary disease* OR artery disease* OR arterial disease* OR heart attack* OR heart failure* OR cardiac failure* OR high blood pressure* OR hypertensi* OR heart patient* OR cerebrovascular disease* OR cerebrovascular disorder* OR vein thrombos* OR embolism* OR stroke* OR cerebrovascular accident* OR cva OR cvas OR vascular accident* OR apoplexy* OR brain infarction*) OR AB (asthma* OR copd OR chronic respiratory disease* OR chronic obstructed pulmonary disease* OR chronic obstructive airway disease* OR chronic obstructive lung disease* OR chronic bronchitis OR emphysema OR coad OR chronic airflow obstruction* OR diabetes OR diabetic* OR dm2 OR niddm OR dm 2 OR t2d* OR dm type 2 OR dm type II OR dm1 OR iddm OR dm 1 OR t1d* OR dm type 1 OR dm type I OR cardiovascular disease* OR cardiovascular disorder* OR cardiovascular disturbance* OR cardiovascular lesion* OR cardiovascular syndrome* OR cvd OR myocardial ischem* OR myocardial infarct* OR heart disease* OR coronary disease* OR artery disease* OR arterial disease* OR heart attack* OR heart failure* OR cardiac failure* OR high blood pressure* OR hypertensi* OR heart patient* OR cerebrovascular disease* OR cerebrovascular disorder* OR vein thrombos* OR embolism* OR stroke* OR cerebrovascular accident* OR cva OR cvas OR vascular accident* OR apoplex* OR brain infarction*) | 107981 |
| S3 | DE "Decision Support Systems" OR TI (tool* OR aid OR aids OR intervention* OR support* OR instrument*) OR AB (tool* OR aid OR aids OR intervention* OR support* OR instrument*) | 1030616 |
| S2 | DE ("Client Participation") OR TI (share* OR sharing OR patient centered* OR patient centred* OR patient focused OR sdm OR prefer*) OR AB (share* OR sharing OR patient centered* OR patient centred* OR patient focused OR sdm OR prefer*) | 252345 |
| S1 | DE "Decision Making" OR TI decision* OR AB decision* | 190151 |

##

## Web of Science History November 7, 2017(read from bottom up)

| Search | Query | Items found |
| --- | --- | --- |
| #6 | #1 AND #2 AND #3 AND #4 AND #5 | 1621 |
| #5 | TS = (rct OR random* OR control* OR trial OR placebo* OR compar* OR group OR groups OR therapy OR treatment OR intervention OR “research design” OR comparative OR “evaluation stud*” OR “follow-up stud*” OR prospective ” OR “single blind” OR “double blind” OR “trebl* blind” OR “triple blind” OR factorial OR allocat* OR assign* OR volunteer* OR crossover OR “cross over”) | 16207865 |
| #4 | TS=(asthma OR "chronic obstructed pulmonary disease*" OR copd OR “chronic respiratory disease*” OR “chronic obstructive airway disease*” OR “chronic obstructive lung disease*” OR “chronic bronchitis” OR emphysema OR coad OR “chronic airflow obstruction*” OR diabetes OR diabetic* OR dm2 OR niddm OR “dm 2” OR t2d* OR “dm type 2” OR “dm type II” OR dm1 OR iddm OR “dm 1” OR “t1d*” OR “dm type 1” OR “dm type I” OR “cardiovascular disease*” OR “cardiovascular disorder*” OR “cardiovascular disturbance*” OR “cardiovascular lesion*” OR “cardiovascular syndrome*” OR cvd OR “myocardial ischem*” OR “myocardial infarct*” OR “heart disease*” OR “coronary disease*” OR “artery disease*” OR “arterial disease*” OR “heart attack*” OR “heart failure*” OR “cardiac failure*” OR “high blood pressure*” OR hypertensi* OR “heart patient*” OR “cerebrovascular disease*” OR “cerebrovascular disorder*” OR “vein thrombos*” OR embolism* OR stroke* OR “cerebrovascular accident*” OR cva OR cvas OR “vascular accident*” OR apoplexy OR “brain infarction*”) | 2149207 |
| #3 | TS =(tool* OR aid OR aids OR intervention* OR support* OR instrument*) | 4183585 |
| #2 | TS=(share* OR sharing OR “patient centered*” OR “patient centred” OR “patient focused” OR sdm OR prefer*) | 1099732 |
| #1 | TS = (Decision*) | 608628 |

##

## The Cochrane Library (Wiley) History November 7, 2017 (read from bottom up)

| Search | Query | Items found |
| --- | --- | --- |
| #6 | #1 AND #2 AND #3 AND #4 AND #5 | 288 |
| #5 | rct OR random* OR control* OR trial OR placebo* OR compar* OR therapy OR treatment OR intervention* OR “research design” OR comparative OR (evaluation NEAR/3 stud*) OR “follow-up stud*” OR prospective OR “single blind” OR “double blind” OR “trebl* blind” OR “triple blind” OR factorial OR allocat* OR assign* OR volunteer* OR crossover OR “cross over” | 1015532 |
| #4 | asthma OR "chronic obstructed pulmonary disease*" OR copd OR “chronic respiratory disease*” OR “chronic obstructive airway disease*” OR “chronic obstructive lung disease*” OR “chronic bronchitis” OR emphysema OR coad OR “chronic airflow obstruction*” OR diabetes OR diabetic* OR dm2 OR niddm OR “dm 2” OR t2d* OR “dm type 2” OR “dm type II” OR dm1 OR iddm OR “dm 1” OR “t1d*” OR “dm type 1” OR “dm type I” OR “cardiovascular disease*” OR “cardiovascular disorder*” OR “cardiovascular disturbance*” OR “cardiovascular lesion*” OR “cardiovascular syndrome*” OR cvd OR “myocardial ischem*” OR “myocardial infarct*” OR “heart disease*” OR “coronary disease*” OR “artery disease*” OR “arterial disease*” OR “heart attack*” OR “heart failure*” OR “cardiac failure*” OR “high blood pressure*” OR hypertensi* OR “heart patient*” OR “cerebrovascular disease*” OR “cerebrovascular disorder*” OR “vein thrombos*” OR embolism* OR stroke* OR “cerebrovascular accident*” OR cva OR cvas OR “vascular accident*” OR apoplexy OR “brain infarction” :ti,ab,kw (Word variations have been searched) | 219598 |
| #3 | tool* OR aid OR aids OR intervention* OR support* OR instrument*:ti,ab,kw (Word variations have been searched) | 240742 |
| #2 | share* OR sharing OR “patient centered*” OR “patient centred” OR “patient focused” OR sdm OR prefer*:ti,ab,kw (Word variations have been searched) | 28117 |
| #1 | decision*:ti,ab,kw (Word variations have been searched) | 20419 |
