# Supplementary material for: Decision aids that facilitate elements of shared decision making in chronic illnesses: a systematic review
Source: Syst Rev. 2019 May 20;8:121. doi: 10.1186/s13643-019-1034-4 (PMC6528254; doi:10.1186/s13643-019-1034-4)
Supplement: Supplementary file 3 — Risk of bias assessment. (DOCX 46 kb) [file 13643_2019_1034_MOESM3_ESM.docx]

**Additional file 3 risk of bias assessment**

| **Study** | **Outcome** | **Risk of bias assessment**^a,b^ | | | | | | | |
| --- | --- | --- | --- | --- | --- | --- | --- | --- | --- |
|  |  | **RSG** | **AC** | **BPP**^c^ | **BOA**^c^ | **IOD** | **SR** | **OB** | **Summary assessment of risk of bias** |
| Knops et al. 2014^1^ |  |  |  |  |  |  |  |  |  |
|  | Decisional conflict | **+** | **?** | **-** | **?** | **-** | **+** | **?** | **High risk of bias** |
|  | Knowledge | **+** | **?** | **-** | **?** | **+** | **+** | **?** | **Unclear risk of bias** |
|  | Anxiety | **+** | **?** | **-** | **?** | **+** | **+** | **?** | **Unclear risk of bias** |
|  | Conversation satisfaction | **+** | **?** | **-** | **?** | **-** | **+** | **?** | **High risk of bias** |
|  | Quality of life | **+** | **?** | **-** | **?** | **+** | **+** | **?** | **Unclear risk of bias** |
|  | Treatment decision (preference) | **+** | **?** | **-** | **?** | **+** | **+** | **?** | **Unclear risk of bias** |
| Man-Son-Hing et al. 1999^2^ |  |  |  |  |  |  |  |  |  |
|  | Decisional conflict | **+** | **+** | **-** | **?** | **?** | **?** | **?** | **Unclear risk of bias** |
|  | Satisfaction with the decision making process | **+** | **+** | **-** | **?** | **?** | **?** | **?** | **Unclear risk of bias** |
|  | Patient participation in decision making | **+** | **+** | **-** | **?** | **?** | **?** | **?** | **Unclear risk of bias** |
|  | Proportion undecided | **+** | **+** | **-** | **?** | **?** | **?** | **?** | **Unclear risk of bias** |
|  | Treatment decision (preference) | **+** | **+** | **-** | **?** | **?** | **?** | **?** | **Unclear risk of bias** |
|  | Adherence | **+** | **+** | **-** | **?** | **?** | **?** | **?** | **Unclear risk of bias** |
| Thomson et al. 2007^3^ |  |  |  |  |  |  |  |  |  |
|  | Decisional conflict | **+** | **?** | **-** | **?** | **-** | **+** | **-** | **High risk of bias** |
|  | Knowledge | **+** | **?** | **-** | **?** | **-** | **+** | **-** | **High risk of bias** |
|  | Anxiety | **+** | **?** | **-** | **?** | **-** | **+** | **-** | **High risk of bias** |
|  | Treatment decision (preference) | **+** | **?** | **-** | **?** | **-** | **+** | **-** | **High risk of bias** |
| Fraenkel et al. 2012^4^ |  |  |  |  |  |  |  |  |  |
|  | Anxiety | **?** | **?** | **-** | **+** | **?** | **?** | **+** | **Unclear risk of bias** |
| Nannenga et al. 2009^5^ |  |  |  |  |  |  |  |  |  |
|  | Decisional conflict | **+** | **+** | **-** | **?** | **?** | **-** | **+** | **High risk of bias** |
|  | Knowledge | **+** | **+** | **-** | **?** | **?** | **-** | **+** | **High risk of bias** |
|  | Conversation duration | **+** | **+** | **-** | **-** | **?** | **-** | **+** | **High risk of bias** |
|  | Patient participation in decision making | **+** | **+** | **-** | **-** | **?** | **-** | **+** | **High risk of bias** |
|  | Trust in physician | **+** | **+** | **-** | **?** | **?** | **-** | **+** | **High risk of bias** |
| Mathers et al. 2012^6^ |  |  |  |  |  |  |  |  |  |
|  | Decisional conflict | **+** | **-** | **-** | **-** | **?** | **+** | **+** | **High risk of bias** |
|  | Conversation duration | **+** | **-** | **-** | **-** | **?** | **+** | **+** | **High risk of bias** |
|  | Proportion undecided | **+** | **-** | **-** | **-** | **?** | **+** | **+** | **High risk of bias** |
|  | Glycemic control | **+** | **-** | **-** | **-** | **?** | **+** | **+** | **High risk of bias** |
| Heisler et al. 2014^7^ |  |  |  |  |  |  |  |  |  |
|  | Decisional conflict | **+** | **+** | **-** | **+** | **?** | **+** | **+** | **Unclear risk of bias** |
|  | Knowledge | **+** | **+** | **-** | **+** | **?** | **+** | **+** | **Unclear risk of bias** |
|  | Illness distress | **+** | **+** | **-** | **+** | **?** | **+** | **+** | **Unclear risk of bias** |
|  | Adherence | **+** | **+** | **-** | **+** | **?** | **+** | **+** | **Unclear risk of bias** |
|  | Glycemic control | **+** | **+** | **-** | **+** | **?** | **+** | **+** | **Unclear risk of bias** |
|  | Diabetes care self-efficacy | **+** | **+** | **-** | **+** | **?** | **+** | **+** | **Unclear risk of bias** |
| Thomas et al. 2013^8^ |  |  |  |  |  |  |  |  |  |
|  | Decisional conflict | **+** | **+** | **-** | **?** | **?** | **+** | **+** | **Unclear risk of bias** |
|  | Knowledge | **+** | **+** | **-** | **?** | **?** | **+** | **+** | **Unclear risk of bias** |
|  | Treatment decision (preference) | **+** | **+** | **-** | **?** | **?** | **+** | **+** | **Unclear risk of bias** |
| Bailey et al. 2016^9^ |  |  |  |  |  |  |  |  |  |
|  | Decisional conflict | **?** | **+** | **-** | **+** | **+** | **+** | **+** | **Unclear risk of bias** |
|  | Knowledge | **?** | **+** | **-** | **+** | **+** | **+** | **+** | **Unclear risk of bias** |
|  | Decision self-efficacy | **?** | **+** | **-** | **+** | **+** | **+** | **+** | **Unclear risk of bias** |
| Perestelo-Perez et al. 2016^10^ |  |  |  |  |  |  |  |  |  |
|  | Decisional conflict | **+** | **-** | **-** | **?** | **?** | **+** | **+** | **High risk of bias** |
|  | Anxiety | **+** | **-** | **-** | **?** | **?** | **+** | **+** | **High risk of bias** |
|  | Satisfaction with the decision making process | **+** | **-** | **-** | **?** | **?** | **+** | **+** | **High risk of bias** |
|  | Conversation duration | **+** | **-** | **-** | **-** | **-** | **+** | **+** | **High risk of bias** |
|  | Illness distress | **+** | **-** | **-** | **?** | **-** | **+** | **+** | **High risk of bias** |
| Slok et al. 2016^11^ |  |  |  |  |  |  |  |  |  |
|  | Quality of life | **+** | **-** | **-** | **+** | **-** | **-** | **+** | **High risk of bias** |
|  | Treatment satisfaction | **+** | **-** | **-** | **+** | **-** | **-** | **+** | **High risk of bias** |
|  | Health status | **+** | **-** | **-** | **+** | **-** | **-** | **+** | **High risk of bias** |
| Denig et al. 2014^12^ |  |  |  |  |  |  |  |  |  |
|  | Quality of life | **+** | **?** | **-** | **?** | **+** | **+** | **-** | **High risk of bias** |
|  | Illness distress | **+** | **?** | **-** | **?** | **+** | **+** | **-** | **High risk of bias** |
|  | Smoking status | **+** | **?** | **-** | **?** | **+** | **+** | **-** | **High risk of bias** |
|  | Treatment satisfaction | **+** | **?** | **-** | **?** | **+** | **+** | **-** | **High risk of bias** |
|  | Diabetes care self-efficacy | **+** | **?** | **-** | **?** | **+** | **+** | **-** | **High risk of bias** |
| Huang et al. 2017^13^ |  |  |  |  |  |  |  |  |  |
|  | Decisional conflict | **?** | **-** | **-** | **?** | **?** | **+** | **+** | **High risk of bias** |
|  | Treatment decision (preference) | **?** | **-** | **-** | **?** | **?** | **+** | **+** | **High risk of bias** |
| El-Jawahri et al. 2016^14^ |  |  |  |  |  |  |  |  |  |
|  | Knowledge | **+** | **+** | **-** | **-** | **?** | **-** | **+** | **High risk of bias** |
|  | Proportion undecided | **+** | **+** | **-** | **-** | **?** | **-** | **+** | **High risk of bias** |
| Morgan et al. 2000^15^ |  |  |  |  |  |  |  |  |  |
|  | Knowledge | **+** | **-** | **-** | **?** | **-** | **?** | **+** | **High risk of bias** |
|  | Satisfaction with the decision making process | **+** | **-** | **-** | **?** | **-** | **?** | **+** | **High risk of bias** |
|  | Treatment decision (preference) | **+** | **-** | **-** | **?** | **-** | **?** | **+** | **High risk of bias** |
| Mullan et al. 2009^16^ |  |  |  |  |  |  |  |  |  |
|  | Decisional conflict | **+** | **-** | **-** | **?** | **?** | **+** | **+** | **High risk of bias** |
|  | Patient participation in decision making | **+** | **-** | **-** | **?** | **?** | **+** | **+** | **High risk of bias** |
|  | Trust in physician | **+** | **-** | **-** | **?** | **?** | **+** | **+** | **High risk of bias** |
|  | Treatment decision (preference) | **+** | **-** | **-** | **?** | **?** | **+** | **+** | **High risk of bias** |
|  | Glycemic control | **+** | **-** | **-** | **?** | **?** | **+** | **+** | **High risk of bias** |
|  | Health status | **+** | **-** | **-** | **?** | **?** | **+** | **+** | **High risk of bias** |
| Mann et al. 2010^17^ |  |  |  |  |  |  |  |  |  |
|  | Decisional conflict | **?** | **?** | **-** | **?** | **?** | **?** | **?** | **Unclear risk of bias** |
| Karagiannis et al. 2016^18^ |  |  |  |  |  |  |  |  |  |
|  | Decisional conflict | **+** | **-** | **-** | **-** | **?** | **+** | **+** | **High risk of bias** |
|  | Knowledge | **+** | **-** | **-** | **-** | **?** | **+** | **+** | **High risk of bias** |
|  | Glycemic control | **+** | **-** | **-** | **-** | **-** | **+** | **+** | **High risk of bias** |
|  | BMI | **+** | **-** | **-** | **-** | **-** | **+** | **+** | **High risk of bias** |
| Coylewright et al. 2016^19^ |  |  |  |  |  |  |  |  |  |
|  | Decisional conflict | **+** | **+** | **-** | **-** | **?** | **-** | **+** | **High risk of bias** |
|  | Patient participation in decision making | **+** | **+** | **-** | **-** | **-** | **-** | **+** | **High risk of bias** |
| Den Ouden et al. 2017^20^ |  |  |  |  |  |  |  |  |  |
|  | Glycemic control | **+** | **-** | **-** | **?** | **?** | **-** | **+** | **High risk of bias** |
|  | Blood pressure | **+** | **-** | **-** | **?** | **?** | **-** | **+** | **High risk of bias** |
|  | Total cholesterol | **+** | **-** | **-** | **?** | **?** | **-** | **+** | **High risk of bias** |
|  | BMI | **+** | **-** | **-** | **?** | **-** | **-** | **+** | **High risk of bias** |
| Gagné et al. 2017^21^ |  |  |  |  |  |  |  |  |  |
|  | Decisional conflict | **+** | **+** | **-** | **+** | **+** | **+** | **+** | **Low risk of bias** |
|  | Knowledge | **+** | **+** | **-** | **+** | **+** | **+** | **+** | **Low risk of bias** |
|  | Achieving treatment goals (asthma control) | **+** | **+** | **-** | **+** | **+** | **+** | **+** | **Low risk of bias** |
|  | Adherence | **+** | **+** | **-** | **+** | **+** | **+** | **+** | **Low risk of bias** |
| Korteland et al. 2017^22^ |  |  |  |  |  |  |  |  |  |
|  | Decisional conflict | **+** | **+** | **-** | **?** | **+** | **+** | **?** | **Unclear risk of bias** |
|  | Anxiety | **+** | **+** | **-** | **?** | **+** | **+** | **?** | **Unclear risk of bias** |
| McAlister et al. 2005^23^ |  |  |  |  |  |  |  |  |  |
|  | Decisional conflict | **+** | **+** | **-** | **+** | **?** | **-** | **+** | **High risk of bias** |
| Weymiller et al. 2007^24^ |  |  |  |  |  |  |  |  |  |
|  | Decisional conflict | **+** | **+** | **-** | **?** | **?** | **-** | **+** | **High risk of bias** |
|  | Treatment decision (preference) | **+** | **+** | **-** | **?** | **?** | **-** | **+** | **High risk of bias** |
|  | Adherence | **+** | **+** | **-** | **?** | **?** | **-** | **+** | **High risk of bias** |

^1^RSG = random sequence generation; AC = allocation concealment; BPP = blinding of participants and personnel; BOA = blinding of outcome assessment; IOD = incomplete outcome data; SR = selective reporting; OB = other bias
^2^+ = Low risk of bias; - = high risk of bias; ? = unclear risk of bias ^3^This element is not taken into account in the summary assessment of risk of bias

**References**

1. Knops A, Goossens A, Ubbink D, Balm R, Koelemay M, Vahl A, et al. A decision aid regarding treatment options for patients with an asymptomatic abdominal aortic aneurysm: A randomised clinical trial. European Journal of Vascular and Endovascular Surgery. 2014;48(3):276-83.

2. Man-Son-Hing M, Laupacis A, O'Connor AM, Biggs J, Drake E, Yetisir E, et al. A patient decision aid regarding antithrombotic therapy for stroke prevention in atrial fibrillation: A randomized controlled trial. JAMA: Journal of the American Medical Association. 1999;282(8):737-43.

3. Thomson RG, Eccles MP, Steen IN, Greenaway J, Stobbart L, Murtagh MJ, et al. A patient decision aid to support shared decision-making on anti-thrombotic treatment of patients with atrial fibrillation: Randomised controlled trial. BMJ Quality & Safety. 2007;16(3):216-23.

4. Fraenkel L, Street RL, Towle V, O'leary JR, Iannone L, Ness PH, et al. A pilot randomized controlled trial of a decision support tool to improve the quality of communication and decision‐making in individuals with atrial fibrillation. Journal of the American Geriatrics Society. 2012;60(8):1434-41.

5. Nannenga MR, Montori VM, Weymiller AJ, Smith SA, Christianson TJ, Bryant SC, et al. A treatment decision aid may increase patient trust in the diabetes specialist. The Statin Choice randomized trial. Health Expectations. 2009;12(1):38-44.

6. Mathers N, Ng CJ, Campbell MJ, Colwell B, Brown I, Bradley A. Clinical effectiveness of a patient decision aid to improve decision quality and glycaemic control in people with diabetes making treatment choices: A cluster randomised controlled trial (PANDAs) in general practice. BMJ Open. 2012;2(6):e001469.

7. Heisler M, Choi H, Palmisano G, Mase R, Richardson C, Fagerlin A, et al. Comparison of community health worker-led diabetes medication decision-making support for low-income Latino and African American adults with diabetes using e-health tools versus print materials: A randomized, controlled trial. Annals of Internal Medicine. 2014;161(10_Supplement):S13-S22.

8. Thomas KL, Zimmer LO, Dai D, Al-Khatib SM, LaPointe NMA, Peterson ED. Educational videos to reduce racial disparities in ICD therapy via innovative designs (VIVID): A randomized clinical trial. American Heart Journal. 2013;166(1):157-63.

9. Bailey RA, Pfeifer M, Shillington AC, Harshaw Q, Funnell MM, VanWingen J, et al. Effect of a patient decision aid (PDA) for type 2 diabetes on knowledge, decisional self-efficacy, and decisional conflict. BMC Health Services Research. 2016;16(1):10.

10. Perestelo-Pérez L, Rivero-Santana A, Boronat M, Sánchez-Afonso JA, Pérez-Ramos J, Montori VM, et al. Effect of the statin choice encounter decision aid in Spanish patients with type 2 diabetes: A randomized trial. Patient Education and Counseling. 2016;99(2):295-9.

11. Slok AH, Kotz D, van Breukelen G, Chavannes NH, Rutten-van Mölken MP, Kerstjens HA, et al. Effectiveness of the Assessment of Burden of COPD (ABC) tool on health-related quality of life in patients with COPD: A cluster randomised controlled trial in primary and hospital care. BMJ Open. 2016;6(7):e011519.

12. Denig P, Schuling J, Haaijer-Ruskamp F, Voorham J. Effects of a patient oriented decision aid for prioritising treatment goals in diabetes: Pragmatic randomised controlled trial. BMJ: British Medical Journal. 2014;349:g5651.

13. Huang ES, Nathan AG, Cooper JM, Lee SM, Shin N, John PM, et al. Impact and feasibility of personalized decision support for older patients with diabetes: A pilot randomized trial. Medical Decision Making. 2017;37(5):611-7.

14. El-Jawahri A, Paasche-Orlow MK, Matlock D, Stevenson LW, Lewis EF, Stewart G, et al. Randomized, controlled trial of an advance care planning video decision support tool for patients with advanced heart failure. Circulation. 2016;134(1):52-60.

15. Morgan MW, Deber RB, Llewellyn‐Thomas HA, Gladstone P, Cusimano R, O'rourke K, et al. Randomized, controlled trial of an interactive videodisc decision aid for patients with ischemic heart disease. Journal of General Internal Medicine. 2000;15(10):685-93.

16. Mullan RJ, Montori VM, Shah ND, Christianson TJ, Bryant SC, Guyatt GH, et al. The diabetes mellitus medication choice decision aid: A randomized trial. Archives of Internal Medicine. 2009;169(17):1560-8.

17. Mann DM, Ponieman D, Montori VM, Arciniega J, McGinn T. The Statin Choice decision aid in primary care: A randomized trial. Patient Education and Counseling. 2010;80(1):138-40.

18. Karagiannis T, Liakos A, Branda ME, Athanasiadou E, Mainou M, Boura P, et al. Use of the Diabetes Medication Choice Decision Aid in patients with type 2 diabetes in Greece: A cluster randomised trial. BMJ Open. 2016;6(11):e012185.

19. Coylewright M, Dick S, Zmolek B, Askelin J, Hawkins E, Branda M, et al. PCI choice decision aid for stable coronary artery disease: A randomized trial. Circulation: Cardiovascular Quality and Outcomes. 2016;9(6):767-76.

20. Den Ouden H, Vos RC, Rutten GE. Effectiveness of shared goal setting and decision making to achieve treatment targets in type 2 diabetes patients: A cluster‐randomized trial (OPTIMAL). Health Expectations. 2017;20(5):1172-80.

21. Gagné ME, Légaré F, Moisan J, Boulet L-P. Impact of adding a decision aid to patient education in adults with asthma: A randomized clinical trial. PloS One. 2017;12(1):e0170055.

22. Korteland NM, Ahmed Y, Koolbergen DR, Brouwer M, de Heer F, Kluin J, et al. Does the use of a decision aid improve decision making in prosthetic heart valve selection?: A multicenter randomized trial. Circulation: Cardiovascular Quality and Outcomes. 2017;10(2):e003178.

23. McAlister FA, Man-Son-Hing M, Straus SE, Ghali WA, Anderson D, Majumdar SR, et al. Impact of a patient decision aid on care among patients with nonvalvular atrial fibrillation: A cluster randomized trial. Canadian Medical Association Journal. 2005;173(5):496-501.

24. Weymiller AJ, Montori VM, Jones LA, Gafni A, Guyatt GH, Bryant SC, et al. Helping patients with type 2 diabetes mellitus make treatment decisions: Statin choice randomized trial. Archives of Internal Medicine. 2007;167(10):1076-82.
