# Supplementary material for: Decision aids that facilitate elements of shared decision making in chronic illnesses: a systematic review
Source: Syst Rev. 2019 May 20;8:121. doi: 10.1186/s13643-019-1034-4 (PMC6528254; doi:10.1186/s13643-019-1034-4)
Supplement: Supplementary file 4 — DA information. (DOCX 77 kb) [file 13643_2019_1034_MOESM4_ESM.docx]

**Additional file 4 DA information**

Information regarding decision aids for cardiovascular diseases

**DA without a name (described in Knops et al. 2014)**

| **DA characteristics** | | | | | | |
| --- | --- | --- | --- | --- | --- | --- |
| **DA target group** | | **Decision to be made** | **Patient decision aid or conversation aid** | **DA format** | **Availability (besides screenshots in articles and/or appendices)** | |
| Patients with asymptomatic abdominal aortic aneurysms | | Elective surgery or watchful waiting | Patient decision aid | Computer-based | [Not](http://www.keuzehulp.info/pp/aaa) available anymore, but the content can be viewed through: <https://sdmstaging.medify.eu/surgery/index_keuzehulp-aneurysma_nl.html> | |
| **SDM elements**^a^ | | | | | | |
| **Situation diagnosis** | | **Choice awareness** | **Option clarification** | **Discussion of harms and benefits** | **Deliberation of patient preferences** | **Making or deferring a decision** |
| **+** | | **+** | **+** | **+** | **+** | **+** |
| **Study information** | | | | | | |
| **Study** | **Country** | **Participants’ condition** | **Setting in which patients are treated (beyond the study)** | **Setting DA use within the study** | **Entire intervention of interest** | **Comparison** |
| Knops et al. 2014^1^ | The Netherlands | Asymptomatic abdominal aortic aneurysm | Secondary/tertiary care | Secondary/tertiary care | Only DA | Usual care |
| **Effects on decisional conflict** | | | | | | |
| **Measurement moment** | | | 1 To 4 weeks post-encounter | | | |
| **Measurement instrument** | | | Decisional Conflict Scale | | | |
| **Effect**^b^ | | | NS | | | |
| **Risk of bias** | | | High risk | | | |
| **Effects on conversation satisfaction** | | | | | | |
| **Measurement moment** | | | 1 To 4 weeks post-encounter | | | |
| **Measurement instrument** | | | Patient Satisfaction Questionnaire | | | |
| **Effect**^b^ | | | NS | | | |
| **Risk of bias** | | | High risk | | | |
| **Effects on treatment decision (preference)** | | | | | | |
| **Measurement moment** | | | 9 To 10 months post-encounter | | | |
| **Measurement instrument** | | | Extracted from the medical record | | | |
| **Effect**^b^ | | | NS^c^ | | | |
| **Risk of bias** | | | Unclear risk | | | |
| **Effects on knowledge** | | | | | | |
| **Measurement moment** | | | 1 To 4 weeks post-encounter | | | |
| **Measurement instrument** | | | 13 Items of the Dutch multiple-choice Aneurysm Knowledge Questionnaire | | | |
| **Effect**^b^ | | | NS | | | |
| **Risk of bias** | | | Unclear risk | | | |
| **Effects on (health-related) quality of life** | | | | | | |
| **Measurement moment** | | | 1 To 4 weeks post-encounter | | | |
| **Measurement instrument** | | | 12-Item Short Form Health Survey (SF-12) | | | |
| **Effect**^b^ | | | NS | | | |
| **Risk of bias** | | | Unclear risk | | | |
| **Effects on anxiety** | | | | | | |
| **Measurement moment** | | | 1 To 4 weeks post-encounter | | | |
| **Measurement instrument** | | | Hospital Anxiety and Depression Scale (HADS) | | | |
| **Effect**^b^ | | | NS | | | |
| **Risk of bias** | | | Unclear risk | | | |

^a^+ = The DA handles the regarding element
^b^+ = Statistical significant effect favoring the DA group; - = statistical significant effect favoring the control group; NS = no statistical significant effect
^c^For this dichotomous outcome is an event regarded as not choosing elective aneurysm impair

**DA without a name (described in Man-Son-Hing et al. 1999)**

| **DA characteristics** | | | | | | | | |
| --- | --- | --- | --- | --- | --- | --- | --- | --- |
| **DA target group** | **Decision to be made** | | **Patient decision aid or conversation aid** | | | **DA format** | **Availability (besides screenshots in articles and/or appendices)** | |
| Patients with atrial fibrillation | Taking aspirin or taking warfarin | | Patient decision aid | | | Both paper-based and audio-based | Not available anymore | |
| **SDM elements**^a^ | | | | | | | | |
| **Situation diagnosis** | | **Choice awareness** | | **Option clarification** | | **Discussion of harms and benefits** | **Deliberation of patient preferences** | **Making or deferring a decision** |
| **?** | | **?** | | **+** | | **+** | **+** | **+** |
| **Study information** | | | | | | | | |
| **Study** | **Country** | | **Participants’ condition** | | **Setting in which patients are treated (beyond the study)** | **Setting DA use within the study** | **Entire intervention of interest** | **Comparison** |
| Man-Son-Hing et al. 1999^2^ | Canada and the United States of America | | Atrial fibrillation | | Unclear | Secondary/tertiary care | Only DA | Usual care |
| **Effects on decisional conflict** | | | | | | | | |
| **Measurement moment** | | | 1 To 4 days post-encounter | | | | | |
| **Measurement instrument** | | | Decisional Conflict Scale and two additional items to elicit patients' perceptions about the extent they were informed about 1) the benefits and risks of warfarin and 2) about benefits and risks of aspirin | | | | | |
| **Effect**^b^ | | | NS | | | | | |
| **Risk of bias** | | | Unclear risk | | | | | |
| **Effects on patient participation in decision making** | | | | | | | | |
| **Measurement moment** | | | 1 To 4 days post-encounter | | | | | |
| **Measurement instrument** | | | 5-Point Likert scale to judge the relative strength of the patient's personal input into the choice versus their physician | | | | | |
| **Effect**^b^ | | | NS^c^ | | | | | |
| **Risk of bias** | | | Unclear risk | | | | | |
| **Effects on satisfaction with the decision making process** | | | | | | | | |
| **Measurement moment** | | | 1 To 4 days post-encounter | | | | | |
| **Measurement instrument** | | | 6 Items using a 5-point Likert scale | | | | | |
| **Effect**^b^ | | | NS | | | | | |
| **Risk of bias** | | | Unclear risk | | | | | |
| **Effects on treatment decision (preference)** | | | | | | | | |
| **Measurement moment** | | | 1 To 4 days post-encounter | | | | | |
| **Measurement instrument** | | | Questionnaire in which participants were, among others, asked what decision regarding antithrombotic therapy was made | | | | | |
| **Effect**^b^ | | | NS^d^ | | | | | |
| **Risk of bias** | | | Unclear risk | | | | | |
| **Effects on proportion undecided** | | | | | | | | |
| **Measurement moment** | | | 1 To 4 days post-encounter | | | | | |
| **Measurement instrument** | | | Participants were asked to indicate whether a decision regarding the choice of antithrombotic therapy had been made in conjunction with their physician | | | | | |
| **Effect**^b^ | | | +^e^ | | | | | |
| **Risk of bias** | | | Unclear risk | | | | | |
| **Effects on adherence** | | | | | | | | |
| **Measurement moment** | | | 6 Months post-encounter | | | | | |
| **Measurement instrument** | | | Participants were asked by telephone which therapy they were currently taking | | | | | |
| **Effect**^b^ | | | NS^f^ | | | | | |
| **Risk of bias** | | | Unclear risk | | | | | |

^a^+ = The DA handles the regarding element; ? = it is doubtful or unclear whether the DA handles the regarding element
^b^+ = Statistical significant effect favoring the DA group; - = statistical significant effect favoring the control group; NS = no statistical significant effect
^c^For this dichotomous outcome is an event regarded as patient reported that he/she made the decision, rather than his/her physician
^d^For this dichotomous outcome is an event regarded as deciding to take warfarin
^e^Odds on not being able to make a definite choice was smaller for the decision aid group compared to the control group
^f^For this dichotomous outcome is an event regarded continuing to take the therapy that was initially chosen

**DA without a name (described in Fraenkel et al. 2012)**

| **DA characteristics** | | | | | | | | | | | |
| --- | --- | --- | --- | --- | --- | --- | --- | --- | --- | --- | --- |
| **DA target group** | | | | **Decision to be made** | | **Patient decision aid or conversation aid** | | **DA format** | | | **Availability (besides screenshots in articles and/or appendices)** |
| Patients with non-valvular atrial fibrillation (NVAF) | | | | Taking aspirin or taking warfarin | | Both patient decision aid and conversation aid | | Both paper-based and computer-based | | | Unclear |
| **SDM elements**^a^ | | | | | | | | | | | |
| **Situation diagnosis** | | **Choice awareness** | | | **Option clarification** | | **Discussion of harms and benefits** | | **Deliberation of patient preferences** | | **Making or deferring a decision** |
| **+** | | **?** | | | **+** | | **+** | | **+** | | **-** |
| **Study information** | | | | | | | | | | | |
| **Study** | **Country** | | | **Participants’ condition** | | **Setting in which patients are treated (beyond the study)** | | **Setting DA use within the study** | | **Entire intervention of interest** | **Comparison** |
| Fraenkel et al. 2012^3^ | The United States of America | | | Non-valvular atrial fibrillation | | Primary care | | Primary care | | Only DA | Unclear |
| **Effects on anxiety** | | | | | | | | | | | |
| **Measurement moment** | | | Immediately post-encounter | | | | | | | | |
| **Measurement instrument** | | | Spielberger State Anxiety Index | | | | | | | | |
| **Effect**^b^ | | | NS | | | | | | | | |
| **Risk of bias** | | | Unclear risk | | | | | | | | |

^a^+ = The DA handles the regarding element; - = the DA does not handle the regarding element
^b^+ = Statistical significant effect favoring the DA group; - = statistical significant effect favoring the control group; NS = no statistical significant effect

**DA without a name (described in Thomas et al. 2013)**

| **DA characteristics** | | | | | | |
| --- | --- | --- | --- | --- | --- | --- |
| **DA target group** | | **Decision to be made** | | **Patient decision aid or conversation aid** | **DA format** | **Availability (besides screenshots in articles and/or appendices)** |
| Individuals eligible for an implantable cardioventer/defibrilator (ICD) | | Whether or not to have an implantable cardioventer/defibrilator (ICD) | | Conversation aid | Computer-based | Not available |
| **SDM elements**^a^ | | | | | | |
| **Situation diagnosis** | **Choice awareness** | **Option clarification** | **Discussion of harms and benefits** | **Deliberation of patient preferences** | | **Making or deferring a decision** |
| **+** | **?** | **+** | **+** | **?** | | **?** |
| **Study information** | | | | | | |
| **Study** | **Country** | **Participants’ condition** | **Setting in which patients are treated (beyond the study)** | **Setting DA use within the study** | **Entire intervention of interest** | **Comparison** |
| Thomas et al. 2013^4^ | The United States of America | Heart failure (class II to III of the New York Heart Association and ejection fraction ≤35%) | Secondary/tertiary care | Secondary/tertiary care | DA + an information sheet | Usual care |
| **Effects on decisional conflict** | | | | | | |
| **Measurement moment** | | 1 Week post-encounter | | | | |
| **Measurement instrument** | | A modified version of the Decisional Conflict Scale | | | | |
| **Effect**^b^ | | NS | | | | |
| **Risk of bias** | | Unclear risk | | | | |
| **Effects on treatment decision (preference)** | | | | | | |
| **Measurement moment** | | 3 Months post-encounter | | | | |
| **Measurement instrument** | | Unclear | | | | |
| **Effect**^b^ | | NS^c^ | | | | |
| **Risk of bias** | | Unclear risk | | | | |
| **Effects on knowledge** | | | | | | |
| **Measurement moment** | | Immediately post-encounter | | | | |
| **Measurement instrument** | | A developed 13-item questionnaire to assess participant's knowledge of SCA, associated risk factors, and ICD therapy | | | | |
| **Effect**^b^ | | NS | | | | |
| **Risk of bias** | | Unclear risk | | | | |

^a^+ = The DA handles the regarding element; ? = it is doubtful or unclear whether the DA handles the regarding element
^b^+ = Statistical significant effect favoring the DA group; - = statistical significant effect favoring the control group; NS = no statistical significant effect
^c^For this dichotomous outcome is an event regarded as ICD implantation within 3 months

**DA without a name (described in El-Jawahri et al. 2016)**

| **DA characteristics** | | | | | | | | | | | | |
| --- | --- | --- | --- | --- | --- | --- | --- | --- | --- | --- | --- | --- |
| **DA target group** | | | | **Decision to be made** | | | | | **Patient decision aid or conversation aid** | | **DA format** | **Availability (besides screenshots in articles and/or appendices)** |
| Patients with advanced heart failure with a limited prognosis | | | | Choosing between three kinds of goals-of-care: life-prolonging care, limited medical care, and comfort care | | | | | Patient decision aid | | Video-based | <https://www.acpdecisions.org/> |
| **SDM elements**^a^ | | | | | | | | | | | | |
| **Situation diagnosis** | | **Choice awareness** | | | **Option clarification** | | **Discussion of harms and benefits** | | | | **Deliberation of patient preferences** | **Making or deferring a decision** |
| **+** | | **+** | | | **+** | | **?** | | | | **?** | **?** |
| **Study information** | | | | | | | | | | | | |
| **Study** | **Country** | | | **Participants’ condition** | | **Setting in which patients are treated (beyond the study)** | | **Setting DA use within the study** | | **Entire intervention of interest** | | **Comparison** |
| El-Jawahri et al. 2016^5^ | The United States of America | | | Advanced heart failure | | Secondary/tertiary care | | Secondary/tertiary care | | DA + listening to a description of the 3 goals of care read out loud by the research assistant | | Listening to the same description of the 3 goals of care used in the video (DA) arm read out loud by the research assistant. |
| **Effects on knowledge** | | | | | | | | | | | | |
| **Measurement moment** | | | Immediately post-encounter | | | | | | | | | |
| **Measurement instrument** | | | 5 True/false items and 1 multiple choice item | | | | | | | | | |
| **Effect**^b^ | | | + | | | | | | | | | |
| **Risk of bias** | | | High risk | | | | | | | | | |

^a^+ = The DA handles the regarding element; ? = it is doubtful or unclear whether the DA handles the regarding element
^b^+ = Statistical significant effect favoring the DA group; - = statistical significant effect favoring the control group; NS = no statistical significant effect

**DA without a name (described in Korteland et al. 2017)**

| **DA characteristics** | | | | | | | |
| --- | --- | --- | --- | --- | --- | --- | --- |
| **DA target group** | | **Decision to be made** | **Patient decision aid or conversation aid** | **DA format** | | | **Availability (besides screenshots in articles and/or appendices)** |
| Patients accepted for elective isolated or combined aortic valve replacement and mitral valve replacement | | Mechanical valve replacement or biological valve replacement | Patient decision aid | Computer-based | | | [www.hartklepkeuze.nl](http://www.hartklepkeuze.nl) |
| **SDM elements**^a^ | | | | | | | |
| **Situation diagnosis** | **Choice awareness** | **Option clarification** | **Discussion of harms and benefits** | **Deliberation of patient preferences** | | **Making or deferring a decision** | |
| **+** | **+** | **+** | **+** | **+** | | **+** | |
| **Study information** | | | | | | | |
| **Study** | **Country** | **Participants’ condition** | **Setting in which patients are treated (beyond the study)** | **Setting DA use within the study** | **Entire intervention of interest** | | **Comparison** |
| Korteland et al. 2017^6^ | The Netherlands | Accepted for elective isolated or combined aortic valve replacement and mitral valve replacement | Secondary/tertiary care | Secondary/tertiary care | Only DA | | Usual care |
| **Effects on decisional conflict** | | | | | | | |
| **Measurement moment** | | Immediately post-encounter | | | | | |
| **Measurement instrument** | | Decisional Conflict Scale^c,d^ | | | | | |
| **Effect**^b^ | | SMD could not be calculated, but there was a non-significant difference in DCS score (median intervention group = 24 (0-69); median control group = 24 (0-72)) | | | | | |
| **Risk of bias** | | Unclear risk | | | | | |
| **Effects on anxiety** | | | | | | | |
| **Measurement moment** | | Immediately post-encounter | | | | | |
| **Measurement instrument** | | The Hospital Anxiety and Depression Scale (HADS)^e,f^ | | | | | |
| **Effect**^b^ | | SMD could not be calculated, but there was a significant difference in HADS score (median intervention group = 6 (0-33); median control group = 9 (0-41)) | | | | | |
| **Risk of bias** | | Unclear risk | | | | | |

^a^+ = The DA handles the regarding element
^b^+ = Statistical significant effect favoring the DA group; - = statistical significant effect favoring the control group; NS = no statistical significant effect
^c^Higher scores indicate less favorable decisional conflict
^d^Scores range from 0 to 100
^e^Higher scores indicate more anxiety
^f^Scores range from 0 to 42

**The Decision Analysis in Routine Treatment Study (DARTS) tool**

| **DA characteristics** | | | | | | | | |
| --- | --- | --- | --- | --- | --- | --- | --- | --- |
| **DA target group** | **Decision to be made** | | | | **Patient decision aid or conversation aid** | **DA format** | **Availability (besides screenshots in articles and/or appendices)** | |
| Patients with atrial fibrillation | Taking aspirin or taking warfarin | | | | Both patient decision aid and conversation aid | Computer –based | The DA is not available in the public domain and is now outdated | |
| **SDM elements**^a^ | | | | | | | | |
| **Situation diagnosis** | | **Choice awareness** | | | **Option clarification** | **Discussion of harms and benefits** | **Deliberation of patient preferences** | **Making or deferring a decision** |
| **?** | | **?** | | | **+** | **+** | **+** | **+** |
| **Study information** | | | | | | | | |
| **Study** | **Country** | | **Participants’ condition** | | **Setting in which patients are treated (beyond the study)** | **Setting DA use within the study** | **Entire intervention of interest** | **Comparison** |
| Thomson et al. 2007^7^ | England | | Atrial fibrillation | | Primary care | Primary care | DA + physician training (in use of the DA) | Decision analysis derived guidelines + physician training (in use of the guideline) |
| **Effects on decisional conflict** | | | | | | | | |
| **Measurement moment** | | | | Immediately post-encounter | | | | |
| **Measurement instrument** | | | | Decisional Conflict Scale | | | | |
| **Effect**^b^ | | | | + | | | | |
| **Risk of bias** | | | | High risk | | | | |
| **Effects on treatment decision (preference)** | | | | | | | | |
| **Measurement moment** | | | | 3 Months post-encounter | | | | |
| **Measurement instrument** | | | | Extracted from the primary care record | | | | |
| **Effect**^b^ | | | | The DA group’s odds on starting or continuing warfarin is significantly lower compared to the control group’s odds | | | | |
| **Risk of bias** | | | | High risk | | | | |
| **Effects on knowledge** | | | | | | | | |
| **Measurement moment** | | | | Immediately post-encounter | | | | |
| **Measurement instrument** | | | | 23 True/false items about atrial fibrillation and stroke | | | | |
| **Effect**^b^ | | | | NS | | | | |
| **Risk of bias** | | | | High risk | | | | |
| **Effects on anxiety** | | | | | | | | |
| **Measurement moment** | | | | Immediately post-encounter | | | | |
| **Measurement instrument** | | | | State Trait Anxiety Inventory (STAI) | | | | |
| **Effect**^b^ | | | | NS | | | | |
| **Risk of bias** | | | | High risk | | | | |

^a^+ = The DA handles the regarding element; ? = it is doubtful or unclear whether the DA handles the regarding element
^b^+ = Statistical significant effect favoring the DA group; - = statistical significant effect favoring the control group; NS = no statistical significant effect

**Ischemic Heart Disease Shared Decision-Making Program (IHD SDP)**

| **DA characteristics** | | | | | | |
| --- | --- | --- | --- | --- | --- | --- |
| **DA target group** | **Decision to be made** | | | **Patient decision aid or conversation aid** | **DA format** | **Availability (besides screenshots in articles and/or appendices)** |
| Patients with ischemic heart disease | Choosing between treatment alternatives for ischemic heart disease: medical therapy, bypass surgery, and angioplasty | | | Unclear | Both computer-based and video-based | Unclear |
| **SDM elements**^a^ | | | | | | |
| **Situation diagnosis** | **Choice awareness** | **Option clarification** | | **Discussion of harms and benefits** | **Deliberation of patient preferences** | **Making or deferring a decision** |
| **+** | **?** | **+** | | **+** | **?** | **?** |
| **Study information** | | | | | | |
| **Study** | **Country** | **Participants’ condition** | **Setting in which patients are treated (beyond the study)** | **Setting DA use within the study** | **Entire intervention of interest** | **Comparison** |
| Morgan et al. 2000^8^ | Canada | Ischemic heart disease | Secondary/tertiary care | Secondary/tertiary care | DA + brochure | Usual care |
| **Effects on satisfaction with the decision making process** | | | | | | |
| **Measurement moment** | | At time of treatment decision | | | | |
| **Measurement instrument** | | A modified version of the 12-item decision making process questionnaire developed by Barry et al. (1997)^9^ | | | | |
| **Effect**^b^ | | NS | | | | |
| **Risk of bias** | | High risk | | | | |
| **Effects on treatment decision (preference)** | | | | | | |
| **Measurement moment** | | Unclear | | | | |
| **Measurement instrument** | | Recorded | | | | |
| **Effect**^b^ | | The DA group’s odds on deciding upon revascularization as initial decision is significantly lower compared to the control group’s odds | | | | |
| **Risk of bias** | | High risk | | | | |
| **Effects on knowledge** | | | | | | |
| **Measurement moment** | | Time of treatment decision | | | | |
| **Measurement instrument** | | 20 True/false items to assess knowledge deemed necessary for an informed treatment decision. This item set was reduced to 15 for patients who were not eligible for angioplasty | | | | |
| **Effect**^b^ | | + | | | | |
| **Risk of bias** | | High risk | | | | |

^a^+ = The DA handles the regarding element; ? = it is doubtful or unclear whether the DA handles the regarding element
^b^+ = Statistical significant effect favoring the DA group; - = statistical significant effect favoring the control group; NS = no statistical significant effect

**PCI choice**

| **DA characteristics** | | | | | | | | |
| --- | --- | --- | --- | --- | --- | --- | --- | --- |
| **DA target group** | **Decision to be made** | | **Patient decision aid or conversation aid** | **DA format** | | **Availability (besides screenshots in articles  and/or appendices)** | | |
| Patients with stable coronary artery disease (SCAD) | Optimal medical therapy (OMT) or percutaneous coronary intervention (PCI) | | Conversation aid | Paper-based | | [https://shareddecisions.mayoclinic.org/ decision-aid-information/decision-aids-for-chronic-disease/pci-choice/](https://shareddecisions.mayoclinic.org/decision-aid-information/decision-aids-for-chronic-disease/pci-choice/) | | |
| **SDM elements**^a^ | | | | | | | | |
| **Situation diagnosis** | **Choice awareness** | **Option clarification** | **Discussion of harms and benefits** | | **Deliberation of patient preferences** | | **Making or deferring a decision** | |
| **-** | **?** | **+** | **+** | | **+** | | **+** | |
| **Study information** | | | | | | | | |
| **Study** | **Country** | **Participants’ condition** | **Setting in which patients are treated (beyond the study)** | **Setting DA use within the study** | | **Entire intervention of interest** | | **Comparison** |
| Coylewright et al. 2016^10^ | The United States of America | Stable coronary artery disease | Secondary/tertiary care | Secondary/tertiary care | | DA + physician training (in use of the DA) | | Usual care |
| **Effects on decisional conflict** | | | | | | | | |
| **Measurement moment** | | Immediately post-encounter | | | | | | |
| **Measurement instrument** | | Decisional Conflict Scale | | | | | | |
| **Effect**^b^ | | NS | | | | | | |
| **Risk of bias** | | High risk | | | | | | |
| **Effects on patient participation in decision making** | | | | | | | | |
| **Measurement moment** | | During the encounter (analyzes based on recorded encounters) | | | | | | |
| **Measurement instrument** | | Observing Patient Involvement in Decision Making Scale (OPTION12) | | | | | | |
| **Effect**^b^ | | NS | | | | | | |
| **Risk of bias** | | High risk | | | | | | |

^a^+ = The DA handles the regarding element; - = the DA does not handle the regarding element
^b^+ = Statistical significant effect favoring the DA group; - = statistical significant effect favoring the control group; NS = no statistical significant effect

**Decision Aid in Atrial Fibrillation (DAAFI)**

| **DA characteristics** | | | | | | |
| --- | --- | --- | --- | --- | --- | --- |
| **DA target group** | **Decision to be made** | **Patient decision aid or conversation aid** | | **DA format** | | **Availability (besides screenshots in articles and/or appendices)** |
| Patients with non-valvular atrial fibrillation (NVAF) | Warfarin, aspirin or no therapy | Patient decision aid | | Both paper-based and audio-based | | Not available anymore |
| **SDM elements**^a^ | | | | | | |
| **Situation diagnosis** | **Choice awareness** | **Option clarification** | **Discussion of harms and benefits** | | **Deliberation of patient preferences** | **Making or deferring a decision** |
| **+** | **+** | **+** | **+** | | **+** | **+** |
| **Study information** | | | | | | |
| **Study** | **Country** | **Participants’ condition** | **Setting in which patients are treated (beyond the study)** | **Setting DA use within the study** | **Entire intervention of interest** | **Comparison** |
| McAlister et al. 2005^11^ | Canada | Non-valvular atrial fibrillation | Primary care | Primary care | Only DA | Usual care |
| **Effects on decisional conflict** | | | | | | |
| **Measurement moment** | | 2 Weeks post-intervention | | | | |
| **Measurement instrument** | | Decisional Conflict Scale | | | | |
| **Effect**^b^ | | + | | | | |
| **Risk of bias** | | High risk | | | | |

^a^+ = The DA handles the regarding element
^b^+ = Statistical significant effect favoring the DA group; - = statistical significant effect favoring the control group; NS = no statistical significant effect

Information regarding decision aids for chronic respiratory diseases

**What are my options regarding inhaled corticosteroids use to improve asthma control? A four-step decision aid / What are my options regarding the combination of inhaled corticosteroids and a long-term action bronchodilator use to improve asthma control? A four-step decision aid**

| **DA characteristics** | | | | | | | | |
| --- | --- | --- | --- | --- | --- | --- | --- | --- |
| **DA target group** | **Decision to be made** | | | | | **Patient decision aid or conversation aid** | **DA format** | **Availability (besides screenshots in articles and/or appendices)** |
| Patients with mild to severe asthma | There are two versions of the decision aid, with both a different decision.  In one DA, patients are asked whether or not they will take their prescribed inhaled corticosteroids to optimize asthma control.  In the other DA, patients are asked whether or not they will take their prescribed inhaled corticosteroids in combination with long-acting β2-agonists to optimize asthma control. | | | | | Both patient decision aid and conversation aid | Paper-based | <https://www.coeurpoumons.ca/> |
| **SDM elements**^a^ | | | | | | | | |
| **Situation diagnosis** | | | **Choice awareness** | **Option clarification** | | **Discussion of harms and benefits** | **Deliberation of patient preferences** | **Making or deferring a decision** |
| **+** | | | **?** | **+** | | **+** | **+** | **+** |
| **Study information** | | | | | | | | |
| **Study** | | **Country** | | **Participants’ condition** | **Setting in which patients are treated (beyond the study)** | **Setting DA use within the study** | **Entire intervention of interest** | **Comparison** |
| Gagné et al. 2017^12^ | | Canada | | Asthma | Secondary/tertiary care | Secondary/tertiary care | DA + patient education | Patient education |
| **Effects on decisional conflict** | | | | | | | | |
| **Measurement moment** | | | | 2 Months post-intervention | | | | |
| **Measurement instrument** | | | | French version of the Decisional Conflict Scale | | | | |
| **Effect**^b^ | | | | NS | | | | |
| **Risk of bias** | | | | Low risk | | | | |
| **Effects on knowledge** | | | | | | | | |
| **Measurement moment** | | | | 2 Months post-intervention | | | | |
| **Measurement instrument** | | | | Questionnaire de connaissances sur l'asthme de langue francaise (QCALF) | | | | |
| **Effect**^b^ | | | | NS | | | | |
| **Risk of bias** | | | | Low risk | | | | |
| **Effects on adherence** | | | | | | | | |
| **Measurement moment** | | | | 2 Months post-intervention | | | | |
| **Measurement instrument** | | | | A 4-item face-to-face interviewer-administered questionnaire | | | | |
| **Effect**^b^ | | | | NS^c^ | | | | |
| **Risk of bias** | | | | Low risk | | | | |
| **Effects on achieving treatment goals** | | | | | | | | |
| **Measurement moment** | | | | 2 Months post-intervention | | | | |
| **Measurement instrument** | | | | The clinical and physiological subscales of the Asthma Control Scoring System (ACSS) | | | | |
| **Effect**^b^ | | | | NS | | | | |
| **Risk of bias** | | | | Low risk | | | | |

^a^+ = The DA handles the regarding element
^b^+ = Statistical significant effect favoring the DA group; - = statistical significant effect favoring the control group; NS = no statistical significant effect
^c^For this dichotomous outcome is an event regarded as appropriate use of pharmacotherapy (asthma drugs). For participants to be considered as appropriate users of asthma drugs, they needed to meet eleven hierarchical criteria, which included using their controller medications for the same number of times every day and at an adequate frequency

**The Assessment of Burden of COPD (ABC) tool**

| **DA characteristics** | | | | | | | | | | |
| --- | --- | --- | --- | --- | --- | --- | --- | --- | --- | --- |
| **DA target group** | **Decision to be made** | | | | **Patient decision aid or conversation aid** | | | **DA format** | **Availability (besides screenshots in articles and/or appendices)** | |
| Patients with chronic obstructive pulmonary disease (COPD) | No specific decision: provides the opportunity to support personalized care planning including a personal treatment goal, and to decide on a treatment plan together through shared decision-making | | | | Conversation aid | | | Computer-based | The DA is available through multiple providers, which can be found through [www.ziektelastmeter.nl](http://www.ziektelastmeter.nl) | |
| **SDM elements**^a^ | | | | | | | | | | |
| **Situation diagnosis** | | **Choice awareness** | | **Option clarification** | | **Discussion of harms and benefits** | | | **Deliberation of patient preferences** | **Making or deferring a decision** |
| **+** | | **?** | | **+** | | **?** | | | **+** | **+** |
| **Study** | | | | | | | | | | |
| **Author** | **Country** | | **Participants’ condition** | | **Setting in which patients are treated (beyond the study)** | | **Setting DA use within the study** | | **Entire intervention of interest** | **Comparison** |
| Slok et al. 2016^13^ | The Netherlands | | Chronic obstructive pulmonary disease (COPD) | | Both primary and secondary/tertiary care | | Both primary and secondary/tertiary care | | Only DA | Usual care |
| **Effects on treatment satisfaction** | | | | | | | | | | |
| **Measurement moment** | | | 12 Months post-encounter | | | | | | | |
| **Measurement instrument** | | | Patient Assessment of Chronic Illness Care (PACIC) | | | | | | | |
| **Effect**^b^ | | | + | | | | | | | |
| **Risk of bias** | | | High risk | | | | | | | |
| **Effects on (health-related) quality of life** | | | | | | | | | | |
| **Measurement moment** | | | 18 Months post-encounter | | | | | | | |
| **Measurement instrument** | | | COPD Assessment Test (CAT) | | | | | | | |
| **Effect**^b^ | | | NS | | | | | | | |
| **Risk of bias** | | | High risk | | | | | | | |
| **Effects on health status** | | | | | | | | | | |
| **Measurement moment** | | | 6 Months post-encounter | | | | | | | |
| **Measurement instrument** | | | St. George's Respiratory Questionnaire (SGRQ) | | | | | | | |
| **Effect**^b^ | | | NS | | | | | | | |
| **Risk of bias** | | | High risk | | | | | | | |

^a^+ = The DA handles the regarding element; ? = it is doubtful or unclear whether the DA handles the regarding element
^b^+ = Statistical significant effect favoring the DA group; - = statistical significant effect favoring the control group; NS = no statistical significant effect

Information regarding decision aids for diabetes

**DA without a name (described in Huang et al. 2017)**

| **DA characteristics** | | | | | | |
| --- | --- | --- | --- | --- | --- | --- |
| **DA target group** | **Decision to be made** | | | **Patient decision aid or conversation aid** | **DA format** | **Availability (besides screenshots in articles and/or appendices)** |
| Patients with type 2 diabetes mellitus (T2DM) | No specific decision: aims to individualize the HbA1c goal | | | Both patient decision aid and conversation aid | Both paper-based and computer-based | Not available anymore |
| **SDM elements**^a^ | | | | | | |
| **Situation diagnosis** | **Choice awareness** | | **Option clarification** | **Discussion of harms and benefits** | **Deliberation of patient preferences** | **Making or deferring a decision** |
| **+** | **+** | | **+** | **+** | **+** | **-** |
| **Study information** | | | | | | |
| **Study** | **Country** | **Participants’ condition** | **Setting in which patients are treated (beyond the study)** | **Setting DA use within the study** | **Entire intervention of interest** | **Comparison** |
| Huang et al. 2017^14^ | The United States of America | Type 2 diabetes mellitus | Secondary/tertiary care | Secondary/tertiary care | DA + physician training (in use of the DA) | Educational brochure |
| **Effects on decisional conflict** | | | | | | |
| **Measurement moment** | | Immediately post-encounter | | | | |
| **Measurement instrument** | | The low literacy version of the Decisional Conflict Scale | | | | |
| **Effect**^b^ | | NS | | | | |
| **Risk of bias** | | High risk | | | | |
| **Effects on treatment decision (preference)** | | | | | | |
| **Measurement moment** | | Immediately post-encounter | | | | |
| **Measurement instrument** | | Physician survey (a change in goal was defined as a 0,5% increase or decrease in HbA1c goal from pre-survey to post-survey responses) | | | | |
| **Effect**^b^ | | NS^c^ | | | | |
| **Risk of bias** | | High risk | | | | |

^a^+ = The DA handles the regarding element; - = the DA does not handle the regarding element
^b^+ = Statistical significant effect favoring the DA group; - = statistical significant effect favoring the control group; NS = no statistical significant effect
^c^For this dichotomous outcome is an event regarded as goal stayed the same

**Statin Choice**

| **DA characteristics** | | | | | | | | | | | | |
| --- | --- | --- | --- | --- | --- | --- | --- | --- | --- | --- | --- | --- |
| **DA target group** | **Decision to be made** | **Patient decision aid or conversation aid** | | | **DA format** | | | | | **Availability (besides screenshots in articles and/or appendices)** | | |
| Patients with type 2 diabetes mellitus (T2DM) | Whether or not to use statins | Conversation aid | | | Paper-based | | | | | [https://statindecisionaid. mayoclinic.org/](https://statindecisionaid.mayoclinic.org/) | | |
| **SDM elements**^a^ | | | | | | | | | | | | |
| **Situation diagnosis** | **Choice awareness** | **Option clarification** | | **Discussion of harms and benefits** | | | | **Deliberation of patient preferences** | | | **Making or deferring a decision** | |
| **+** | **?** | **+** | | **+** | | | | **-** | | | **+** | |
| **Study information** | | | | | | | | | | | | |
| **Studies** | **Country** | **Participants’ condition** | | **Setting in which patients are treated (beyond the study)** | | | **Setting DA use within the study** | | | **Entire intervention of interest** | | **Comparison** |
| Weymiller et al. 2007^15^; Nannenga et al. 2009^16^ (both publications are based on the same RCT) | The United States of America | Type 2 diabetes mellitus | | Secondary/tertiary care | | | Secondary/tertiary care | | | Only DA | | Educational pamphlet |
| Mann et al. 2010^17^ | The United States of America | Diabetes mellitus (any type) | | Primary care | | | Primary care | | | Only DA | | Printed material |
| Perestelo-Perez et al. 2016^18^ | Spain (Spanish version of Statin Choice) | Type 2 diabetes mellitus | | Primary care | | | Primary care | | | DA + physician training (in use of the DA) | | Usual care |
| **Effects on decisional conflict** | | | | | | | | | | | | |
| **Study** | **Nannenga et al. 2009 / Weymiller et al. 2007** | | **Mann et al. 2010** | | | | | | **Perestelo Perez et al. 2016** | | | |
| **Measurement moment** | Immediately post-encounter | | Immediately post-encounter | | | | | | Immediately post-encounter | | | |
| **Measurement instrument** | Decisional Conflict Scale | | Decisional Conflict Scale | | | | | | Decisional Conflict Scale | | | |
| **Effect**^b^ | + | | NS | | | | | | NS | | | |
| **Risk of bias** | High risk | | Unclear risk | | | | | | High risk | | | |
| **Effects on trust in physician** | | | | | | | | | | | | |
| **Study** | **Nannenga et al. 2009** | | | | | | | | | | | |
| **Measurement moment** | Immediately post-encounter | | | | | | | | | | | |
| **Measurement instrument** | Trust in Physician Scale | | | | | | | | | | | |
| **Effect**^b^ | NS | | | | | | | | | | | |
| **Risk of bias** | High risk | | | | | | | | | | | |
| **Effects on patient participation in decision making** | | | | | | | | | | | | |
| **Study** | **Nannenga et al. 2009** | | | | | | | | | | | |
| **Measurement moment** | During the encounter (analyzes based on recorded encounters) | | | | | | | | | | | |
| **Measurement instrument** | Observing Patient Involvement in Decision Making Scale (OPTION12) | | | | | | | | | | | |
| **Effect**^b^ | NS | | | | | | | | | | | |
| **Risk of bias** | High risk | | | | | | | | | | | |
| **Effects on conversation duration** | | | | | | | | | | | | |
| **Study** | **Nannenga et al. 2009** | | | | | **Perestelo Perez et al. 2016** | | | | | | |
| **Measurement moment** | During the encounter | | | | | During the encounter | | | | | | |
| **Measurement instrument** | Videotapes of the encounters | | | | | Documented by the physician | | | | | | |
| **Effect**^b^ | NS | | | | | NS | | | | | | |
| **Risk of bias** | High risk | | | | | High risk | | | | | | |
| **Effects on knowledge** | | | | | | | | | | | | |
| **Study** | **Nannenga et al. 2009** | | | | | | | | | | | |
| **Measurement moment** | Immediately post-encounter | | | | | | | | | | | |
| **Measurement instrument** | 16 Knowledge items | | | | | | | | | | | |
| **Effect**^b^ | NS | | | | | | | | | | | |
| **Risk of bias** | High risk | | | | | | | | | | | |
| **Effects on treatment decision (preference)** | | | | | | | | | | | | |
| **Study** | **Weymiller et al. 2007** | | | | | | | | | | | |
| **Measurement moment** | Immediately post-encounter | | | | | | | | | | | |
| **Measurement instrument** | Percentage of participants not receiving statin therapy at baseline deciding to start statin therapy (unclear whether this is measured based on recordings or by questionnaires) | | | | | | | | | | | |
| **Effect**^b^ | NS^c^ | | | | | | | | | | | |
| **Risk of bias** | High risk | | | | | | | | | | | |
| **Effects on satisfaction with the decision making process** | | | | | | | | | | | | |
| **Study** | **Perestelo Perez et al. 2016** | | | | | | | | | | | |
| **Measurement moment** | Immediately post-encounter | | | | | | | | | | | |
| **Measurement instrument** | A modified version of the 12-item decision making process questionnaire developed by Barry et al. (1995)^19^ | | | | | | | | | | | |
| **Effect**^b^ | + | | | | | | | | | | | |
| **Risk of bias** | High risk | | | | | | | | | | | |
| **Effects on illness distress** | | | | | | | | | | | | |
| **Study** | **Perestelo Perez et al. 2016** | | | | | | | | | | | |
| **Measurement moment** | 3 Months post-encounter | | | | | | | | | | | |
| **Measurement instrument** | Problem Areas In Diabetes (PAID) | | | | | | | | | | | |
| **Effect**^b^ | NS | | | | | | | | | | | |
| **Risk of bias** | High risk | | | | | | | | | | | |
| **Effects on anxiety** | | | | | | | | | | | | |
| **Study** | **Perestelo Perez et al. 2016** | | | | | | | | | | | |
| **Measurement moment** | Immediately post-encounter | | | | | | | | | | | |
| **Measurement instrument** | Spanish version of the State Trait Anxiety Inventory (STAI) | | | | | | | | | | | |
| **Effect**^b^ | NS | | | | | | | | | | | |
| **Risk of bias** | High risk | | | | | | | | | | | |
| **Effects on adherence** | | | | | | | | | | | | |
| **Study** | **Weymiller et al. 2007** | | | | | | | | | | | |
| **Measurement moment** | 3 Months post-encounter | | | | | | | | | | | |
| **Measurement instrument** | Mailed survey (and telephone calls for non-responders) to determine whether participants had missed any doses in the last week | | | | | | | | | | | |
| **Effect**^b^ | NS^d^ | | | | | | | | | | | |
| **Risk of bias** | High risk | | | | | | | | | | | |

^a^+ = The DA handles the regarding element; - = the DA does not handle the regarding element
^b^+ = Statistical significant effect favoring the DA group; - = statistical significant effect favoring the control group; NS = no statistical significant effect
^c^For this dichotomous outcome is an event regarded as deciding not to take statin therapy
^d^For this dichotomous outcome is an event regarded as not missing any dose in the last week

**PANDAs**

| **DA characteristics** | | | | | | | | |
| --- | --- | --- | --- | --- | --- | --- | --- | --- |
| **DA target group** | **Decision to be made** | | **Patient decision aid or conversation aid** | | | **DA format** | **Availability (besides screenshots in articles and/or appendices)** | |
| Patients with type 2 diabetes mellitus | Make no change, lifestyle modification, or insulin therapy | | Both patient decision aid and conversation aid | | | Paper-based | Academic Unit of Primary Medical Care, Faculty of Medicine, University of Sheffield:  <https://www.sheffield.ac.uk/medicine/research/aupmc> | |
| **SDM elements**^a^ | | | | | | | | |
| **Situation diagnosis** | | **Choice awareness** | | **Option clarification** | | **Discussion of harms and benefits** | **Deliberation of patient preferences** | **Making or deferring a decision** |
| **?** | | **?** | | **+** | | **+** | **+** | **?** |
| **Study information** | | | | | | | | |
| **Study** | **Country** | | **Participants’ condition** | | **Setting in which patients are treated (beyond the study)** | **Setting DA use within the study** | **Entire intervention of interest** | **Comparison** |
| Mathers et al. 2012^20^ | United Kingdom | | Type 2 diabetes mellitus | | Primary care | Primary care | DA + physician training (in use of the DA) | Usual care |
| **Effects on decisional conflict** | | | | | | | | |
| **Measurement moment** | | | Immediately post-encounter | | | | | |
| **Measurement instrument** | | | Decisional Conflict Scale | | | | | |
| **Effect**^b^ | | | + | | | | | |
| **Risk of bias** | | | High risk | | | | | |
| **Effects on proportion undecided** | | | | | | | | |
| **Measurement moment** | | | Immediately post-encounter | | | | | |
| **Measurement instrument** | | | Unclear | | | | | |
| **Effect**^b^ | | | NS^c^ | | | | | |
| **Risk of bias** | | | High risk | | | | | |
| **Effects on conversation duration** | | | | | | | | |
| **Measurement moment** | | | During the encounter | | | | | |
| **Measurement instrument** | | | Timed by the researcher from the point the patient entered the consultation room to the time patient left | | | | | |
| **Effect**^b^ | | | NS | | | | | |
| **Risk of bias** | | | High risk | | | | | |
| **Effects on glycemic control** | | | | | | | | |
| **Measurement moment** | | | 6 Months post-encounter | | | | | |
| **Measurement instrument** | | | Reported by the healthcare provider based on latest HbA1c on the medical records | | | | | |
| **Effect**^b^ | | | NS | | | | | |
| **Risk of bias** | | | High risk | | | | | |

^a^+ = The DA handles the regarding element; ? = it is doubtful or unclear whether the DA handles the regarding element
^b^+ = Statistical significant effect favoring the DA group; - = statistical significant effect favoring the control group; NS = no statistical significant effect
^c^For this dichotomous outcome is an event regarded as being undecided

**iDecide (Spanish: iDecido)**

| **DA characteristics** | | | | | | | | |
| --- | --- | --- | --- | --- | --- | --- | --- | --- |
| **DA target group** | | | **Decision to be made** | | | **Patient decision aid or conversation aid** | **DA format** | **Availability (besides screenshots in articles and/or appendices)** |
| Patients with type 2 diabetes mellitus (T2DM) | | | No specific decision: enables navigation by the community health worker and the patient to selectively explore diabetes treatment issues most important to the patient | | | Patient decision aid | Computer-based | Unclear |
| **SDM elements**^a^ | | | | | | | | |
| **Situation diagnosis** | | **Choice awareness** | | **Option clarification** | **Discussion of harms and benefits** | **Deliberation of patient preferences** | **Making or deferring a decision** | |
| **+** | | **?** | | **+** | **+** | **+** | **+** | |
| **Study information** | | | | | | | | |
| **Study** | **Country** | | **Participants’ condition** | **Setting in which patients are treated (beyond the study)** | **Setting DA use within the study** | **Entire intervention of interest** | **Comparison** | |
| Heisler et al. 2014^21^ | The United States of America | | Diabetes mellitus (any type) | Unclear | Location agreed upon with community health worker | DA + community health worker training (in motivational interviewing-based communication approaches and diabetes self-management support) | Booklets (guides) + community health worker training (in motivational interviewing-based communication approaches and diabetes self-management support) | |
| **Effects on decisional conflict** | | | | | | | | |
| **Measurement moment** | | | | Immediately post-encounter | | | | |
| **Measurement instrument** | | | | Decisional Conflict Scale | | | | |
| **Effect**^b^ | | | | NS | | | | |
| **Risk of bias** | | | | Unclear risk | | | | |
| **Effects on knowledge** | | | | | | | | |
| **Measurement moment** | | | | Immediately post-encounter | | | | |
| **Measurement instrument** | | | | Items regarding knowledge about anti-hyperglycemic medications | | | | |
| **Effect**^b^ | | | | NS | | | | |
| **Risk of bias** | | | | Unclear risk | | | | |
| **Effects on diabetes care self-efficacy** | | | | | | | | |
| **Measurement moment** | | | | Immediately post-encounter | | | | |
| **Measurement instrument** | | | | Unclear | | | | |
| **Effect**^b^ | | | | NS | | | | |
| **Risk of bias** | | | | Unclear risk | | | | |
| **Effects on illness distress** | | | | | | | | |
| **Measurement moment** | | | | 3 Months post-encounter | | | | |
| **Measurement instrument** | | | | Diabetes Distress Scale | | | | |
| **Effect**^b^ | | | | + | | | | |
| **Risk of bias** | | | | Unclear risk | | | | |
| **Effects on glycemic control** | | | | | | | | |
| **Measurement moment** | | | | 3 Months post-encounter | | | | |
| **Measurement instrument** | | | | HbA1c in % (measured by the Bayer DCA 2000+ point-of-care analyzer) | | | | |
| **Effect**^b^ | | | | NS | | | | |
| **Risk of bias** | | | | Unclear risk | | | | |
| **Effects on adherence** | | | | | | | | |
| **Measurement moment** | | | | 3 Months post-encounter | | | | |
| **Measurement instrument** | | | | A self-reported measure of medication adherence developed by Morisky et al (1986)^22^ | | | | |
| **Effect**^b^ | | | | NS | | | | |
| **Risk of bias** | | | | Unclear risk | | | | |

^a^+ = The DA handles the regarding element; ? = it is doubtful or unclear whether the DA handles the regarding element
^b^+ = Statistical significant effect favoring the DA group; - = statistical significant effect favoring the control group; NS = no statistical significant effect

**Diabetes Decision Aid for T2DM**

| **DA characteristics** | | | | | | | | | |
| --- | --- | --- | --- | --- | --- | --- | --- | --- | --- |
| **DA target group** | **Decision to be made** | | | | **Patient decision aid or conversation aid** | | **DA format** | **Availability (besides screenshots in articles and/or appendices)** | |
| Patients with type 2 diabetes mellitus (T2DM) | Decisions about anti-hyperglycemic medication intensification for patients for whom first-line treatment with metformin is no longer effective | | | | Patient decision aid | | Computer-based | To preview the DA, or to learn how to use it within individual practices, clinicians must register through <http://www.diabetesdecisionaid.com/>. The DA is being used in integrated delivery networks (IDNs) in the USA. Clinicians in the IDNs have access through their institutions. | |
| **SDM elements**^a^ | | | | | | | | | |
| **Situation diagnosis** | | **Choice awareness** | | **Option clarification** | | **Discussion of harms and benefits** | | **Deliberation of patient preferences** | **Making or deferring a decision** |
| **+** | | **?** | | **+** | | **+** | | **+** | **+** |
| **Study information** | | | | | | | | | |
| **Study** | **Country** | | **Participants’ condition** | | **Setting in which patients are treated (beyond the study)** | | **Setting DA use within the study** | **Entire intervention of interest** | **Comparison** |
| Bailey et al. 2016^23^ | The United States of America | | Type 2 diabetes mellitus | | Both primary and secondary/tertiary care | | Both primary and secondary/tertiary care | DA + physician training (in use of the DA) | Usual care |
| **Effects on decisional conflict** | | | | | | | | | |
| **Measurement moment** | | | 4 To 6 weeks post-intervention | | | | | | |
| **Measurement instrument** | | | Decisional Conflict Scale | | | | | | |
| **Effect**^b^ | | | + | | | | | | |
| **Risk of bias** | | | Unclear risk | | | | | | |
| **Effects on decision self-efficacy** | | | | | | | | | |
| **Measurement moment** | | | 4 To 6 weeks post-intervention | | | | | | |
| **Measurement instrument** | | | Decision Self Efficacy Scale (DSES) | | | | | | |
| **Effect**^b^ | | | + | | | | | | |
| **Risk of bias** | | | Unclear risk | | | | | | |
| **Effects on knowledge** | | | | | | | | | |
| **Measurement moment** | | | 4 To 6 weeks post-intervention | | | | | | |
| **Measurement instrument** | | | A developed questionnaire to assess understanding of how different treatments differ in terms of their impact on glycemic control (amount and durability), impact on weight, risk of hypoglycemia and other adverse events, route of administration, frequency of dose administration and blood glucose monitoring, and financial costs | | | | | | |
| **Effect**^b^ | | | + | | | | | | |
| **Risk of bias** | | | Unclear risk | | | | | | |

^a^+ = The DA handles the regarding element
^b^+ = Statistical significant effect favoring the DA group; - = statistical significant effect favoring the control group; NS = no statistical significant effect

**Patient-oriented treatment decision aid for diabetes (PORTDA-diab)**

| **DA characteristics** | | | | | | | | | | | | |
| --- | --- | --- | --- | --- | --- | --- | --- | --- | --- | --- | --- | --- |
| **DA target group** | **Decision to be made** | | | | **Patient decision aid or conversation aid** | | | **DA format** | | **Availability (besides screenshots in articles and/or appendices)** | | |
| Patients with type 2 diabetes mellitus (T2DM) | No specific decision: stimulates and supports effective interactions between patients and healthcare providers | | | | Both patient decision aid and conversation aid | | | Both paper-based and computer-based | | The computer-based version is not available anymore. The paper-based version is available by contact the first author of the article ([p.denig@umcg.nl](mailto:p.denig@umcg.nl)) | | |
| **SDM elements**^a^ | | | | | | | | | | | | |
| **Situation diagnosis** | | **Choice awareness** | | **Option clarification** | | **Discussion of harms and benefits** | | | **Deliberation of patient preferences** | | **Making or deferring a decision** | |
| **+** | | **+** | | **+** | | **+** | | | **+** | | **+** | |
| **Study information** | | | | | | | | | | | | |
| **Study** | **Country** | | **Participants’ condition** | | **Setting in which patients are treated (beyond the study)** | | **Setting DA use within the study** | | | **Entire intervention of interest** | | **Comparison** |
| Denig et al. 2014^24^ | The Netherlands | | Type 2 diabetes mellitus | | Primary care | | Primary care | | | Only DA | | Usual care |
| **Effects on treatment satisfaction** | | | | | | | | | | | | |
| **Measurement moment** | | | 3 To 4 months post-encounter | | | | | | | | | |
| **Measurement instrument** | | | Patients' Evaluation of Quality of Diabetes care (PEQD) questionnaire | | | | | | | | | |
| **Effect**^b^ | | | NS | | | | | | | | | |
| **Risk of bias** | | | High risk | | | | | | | | | |
| **Effects on (health-related) quality of life** | | | | | | | | | | | | |
| **Measurement moment** | | | 3 To 4 months post-encounter | | | | | | | | | |
| **Measurement instrument** | | | Dutch version of the EuroQol (EQ-5D) | | | | | | | | | |
| **Effect**^b^ | | | NS | | | | | | | | | |
| **Risk of bias** | | | High risk | | | | | | | | | |
| **Effects on illness distress** | | | | | | | | | | | | |
| **Measurement moment** | | | 3 To 4 months post-encounter | | | | | | | | | |
| **Measurement instrument** | | | Problem Areas In Diabetes (PAID) | | | | | | | | | |
| **Effect**^b^ | | | NS | | | | | | | | | |
| **Risk of bias** | | | High risk | | | | | | | | | |
| **Effects on smoking status** | | | | | | | | | | | | |
| **Measurement moment** | | | 6 Months post-encounter | | | | | | | | | |
| **Measurement instrument** | | | Extracted from the medical record | | | | | | | | | |
| **Effect**^b^ | | | NS^c^ | | | | | | | | | |
| **Risk of bias** | | | High risk | | | | | | | | | |

^a^+ = The DA handles the regarding element
^b^+ = Statistical significant effect favoring the DA group; - = statistical significant effect favoring the control group; NS = no statistical significant effect
^c^For this dichotomous outcome is an event regarded as smoking

**Diabetes Medication Choice Decision Aid**

| **DA characteristics** | | | | | | | | | |
| --- | --- | --- | --- | --- | --- | --- | --- | --- | --- |
| **DA target group** | **Decision to be made** | | | | **Patient decision aid or conversation aid** | | **DA format** | **Availability (besides screenshots in articles and/or appendices)** | |
| Patients with type 2 diabetes mellitus (T2DM) | Whether or not adding an anti-hyperglycemic agent, and if choosing to add which option to choose | | | | Conversation aid | | Paper-based | [https://shareddecisions.mayoclinic.org/ decision-aid-information/decision-aids-for-chronic-disease/diabetes-medication-management/](https://shareddecisions.mayoclinic.org/decision-aid-information/decision-aids-for-chronic-disease/diabetes-medication-management/) | |
| **SDM elements**^a^ | | | | | | | | | |
| **Situation diagnosis** | | **Choice awareness** | | **Option clarification** | | **Discussion of harms and benefits** | | **Deliberation of patient preferences** | **Making or deferring a decision** |
| **-** | | **?** | | **+** | | **+** | | **+** | **-** |
| **Study information** | | | | | | | | | |
| **Studies** | **Country** | | **Participants’ condition** | | **Setting in which patients are treated (beyond the study)** | | **Setting DA use within the study** | **Entire intervention of interest** | **Comparison** |
| Mullan et al. 2009^25^ | The United States of America | | Type 2 diabetes mellitus | | Primary care | | Primary care | DA + physician training (in use of the DA) | Pamphlet |
| Karagiannis et al. 2016^26^ | Greece | | Type 2 diabetes mellitus | | Both primary and secondary/tertiary care | | Both primary and secondary/tertiary care | DA + physician training (in use of the DA) | Usual care |
| **Effects on decisional conflict** | | | | | | | | | |
| **Study** | | | **Mullan et al. 2009** | | | | | **Karagiannis et al. 2016** | |
| **Measurement moment** | | | Immediately post-encounter | | | | | Immediately post-encounter | |
| **Measurement instrument** | | | Decisional Conflict Scale | | | | | 13-Item modified version of the Decisional Conflict Scale | |
| **Effect**^b^ | | | NS | | | | | NS | |
| **Risk of bias** | | | High risk | | | | | High risk | |
| **Effects on trust in physician** | | | | | | | | | |
| **Study** | | | **Mullan et al. 2009** | | | | | | |
| **Measurement moment** | | | Immediately post-encounter | | | | | | |
| **Measurement instrument** | | | 9-Item version of the Trust in Physician Scale | | | | | | |
| **Effect**^b^ | | | NS | | | | | | |
| **Risk of bias** | | | High risk | | | | | | |
| **Effects on patient participation in decision making** | | | | | | | | | |
| **Study** | | | **Mullan et al. 2009** | | | | | | |
| **Measurement moment** | | | During the encounter | | | | | | |
| **Measurement instrument** | | | Observing Patient Involvement in Decision Making Scale (OPTION12) | | | | | | |
| **Effect**^b^ | | | + | | | | | | |
| **Risk of bias** | | | High risk | | | | | | |
| **Effects on treatment decision preference** | | | | | | | | | |
| **Study** | | | **Mullan et al. 2009** | | | | | | |
| **Measurement moment** | | | Immediately post-encounter | | | | | | |
| **Measurement instrument** | | | Physician survey | | | | | | |
| **Effect**^b^ | | | NS^c^ | | | | | | |
| **Risk of bias** | | | High risk | | | | | | |
| **Effects on knowledge** | | | | | | | | | |
| **Study** | | | **Karagiannis et al. 2016** | | | | | | |
| **Measurement moment** | | | Immediately post-encounter | | | | | | |
| **Measurement instrument** | | | 6-Item questionnaire addressing general knowledge about T2DM management and medications | | | | | | |
| **Effect**^b^ | | | NS | | | | | | |
| **Risk of bias** | | | High risk | | | | | | |
| **Effects on health status** | | | | | | | | | |
| **Study** | | | **Mullan et al. 2009** | | | | | | |
| **Measurement moment** | | | 6 Months post-encounter | | | | | | |
| **Measurement instrument** | | | Asking patients by telephone to rate their health as excellent, very good, good, fair, or poor | | | | | | |
| **Effect**^b^ | | | NS | | | | | | |
| **Risk of bias** | | | High risk | | | | | | |
| **Effects on glycemic control** | | | | | | | | | |
| **Study** | | | **Mullan et al. 2009** | | | | | **Karagiannis et al. 2016** | |
| **Measurement moment** | | | 6 Months post-encounter | | | | | 3 Months post-encounter | |
| **Measurement instrument** | | | HbA1c in % (extracted from the medical record) | | | | | HbA1c in % (measured at a local lab or lab of patient’s choice) | |
| **Effect**^b^ | | | NS | | | | | NS | |
| **Risk of bias** | | | High risk | | | | | High risk | |
| **Effects on BMI** | | | | | | | | | |
| **Study** | | | **Karagiannis et al. 2016** | | | | | | |
| **Measurement moment** | | | 6 Months post-encounter | | | | | | |
| **Measurement instrument** | | | BMI (measured by physician) | | | | | | |
| **Effect**^b^ | | | NS | | | | | | |
| **Risk of bias** | | | High risk | | | | | | |

^a^+ = The DA handles the regarding element; ? = it is doubtful or unclear whether the DA handles the regarding element; - = the DA does not handle the regarding element
^b^+ = Statistical significant effect favoring the DA group; - = statistical significant effect favoring the control group; NS = no statistical significant effect
^c^For this dichotomous outcome is an event regarded as continue taking current medications

**OPTIMAL**

| **DA characteristics** | | | | | | | | |
| --- | --- | --- | --- | --- | --- | --- | --- | --- |
| **DA target group** | **Decision to be made** | | | **Patient decision aid or conversation aid** | | | **DA format** | **Availability (besides screenshots in articles and/or appendices)** |
| Patients with type 2 diabetes mellitus (T2DM) | Two decisions: 1) regular treatment or intensive treatment and 2) prioritizing treatment targets (HbA1c, cholesterol, blood pressure, body weight, smoking habits) | | | Conversation aid | | | Paper-based | Not available |
| **SDM elements**^a^ | | | | | | | | |
| **Situation diagnosis** | | **Choice awareness** | **Option clarification** | **Discussion of harms and benefits** | | **Deliberation of patient preferences** | | **Making or deferring a decision** |
| **-** | | **?** | **+** | **+** | | **+** | | **+** |
| **Study information** | | | | | | | | |
| **Study** | **Country** | | **Participants’ condition** | **Setting in which patients are treated (beyond the study)** | **Setting DA use within the study** | | **Entire intervention of interest** | **Comparison** |
| den Ouden et al. 2017^27^ | The Netherlands | | Type 2 diabetes mellitus | Primary care | Primary care | | Only DA | Usual care |
| **Effects on glycemic control** | | | | | | | | |
| **Measurement moment** | | | 24 Months post-encounter | | | | | |
| **Measurement instrument** | | | HbA1c in mmol/mol (measured by a high-performance liquid chromatography (Tosoh G8 machine)) | | | | | |
| **Effect**^b^ | | | NS | | | | | |
| **Risk of bias** | | | High risk | | | | | |
| **Effects on total cholesterol** | | | | | | | | |
| **Measurement moment** | | | 24 Months post-encounter | | | | | |
| **Measurement instrument** | | | Total cholesterol in mmol/L (measured by standard enzymatic techniques (Cobas 8000 machine)) | | | | | |
| **Effect**^b^ | | | NS | | | | | |
| **Risk of bias** | | | High risk | | | | | |
| **Effects on blood pressure** | | | | | | | | |
| **Measurement moment** | | | 24 Months post-encounter | | | | | |
| **Measurement instrument** | | | Systolic blood pressure in mm Hg (measured by two measurements after at least 10 minutes rest while participants were seated with the cuff on the predominant arm at the level of the heart) | | | | | |
| **Effect**^b^ | | | NS | | | | | |
| **Risk of bias** | | | High risk | | | | | |
| **Effects on BMI** | | | | | | | | |
| **Measurement moment** | | | 24 Months post-encounter | | | | | |
| **Measurement instrument** | | | BMI (measured by general practitioner) | | | | | |
| **Effect**^b^ | | | NS | | | | | |
| **Risk of bias** | | | High risk | | | | | |

^a^+ = The DA handles the regarding element; - = the DA does not handle the regarding element
^b^+ = Statistical significant effect favoring the DA group; - = statistical significant effect favoring the control group; NS = no statistical significant effect

**References**

1. Knops A, Goossens A, Ubbink D, Balm R, Koelemay M, Vahl A, et al. A decision aid regarding treatment options for patients with an asymptomatic abdominal aortic aneurysm: A randomised clinical trial. European Journal of Vascular and Endovascular Surgery. 2014;48(3):276-83.

2. Man-Son-Hing M, Laupacis A, O'Connor AM, Biggs J, Drake E, Yetisir E, et al. A patient decision aid regarding antithrombotic therapy for stroke prevention in atrial fibrillation: A randomized controlled trial. JAMA: Journal of the American Medical Association. 1999;282(8):737-43.

3. Fraenkel L, Street RL, Towle V, O'leary JR, Iannone L, Ness PH, et al. A pilot randomized controlled trial of a decision support tool to improve the quality of communication and decision‐making in individuals with atrial fibrillation. Journal of the American Geriatrics Society. 2012;60(8):1434-41.

4. Thomas KL, Zimmer LO, Dai D, Al-Khatib SM, LaPointe NMA, Peterson ED. Educational videos to reduce racial disparities in ICD therapy via innovative designs (VIVID): A randomized clinical trial. American Heart Journal. 2013;166(1):157-63.

5. El-Jawahri A, Paasche-Orlow MK, Matlock D, Stevenson LW, Lewis EF, Stewart G, et al. Randomized, controlled trial of an advance care planning video decision support tool for patients with advanced heart failure. Circulation. 2016;134(1):52-60.

6. Korteland NM, Ahmed Y, Koolbergen DR, Brouwer M, de Heer F, Kluin J, et al. Does the use of a decision aid improve decision making in prosthetic heart valve selection?: A multicenter randomized trial. Circulation: Cardiovascular Quality and Outcomes. 2017;10(2):e003178.

7. Thomson RG, Eccles MP, Steen IN, Greenaway J, Stobbart L, Murtagh MJ, et al. A patient decision aid to support shared decision-making on anti-thrombotic treatment of patients with atrial fibrillation: Randomised controlled trial. BMJ Quality & Safety. 2007;16(3):216-23.

8. Morgan MW, Deber RB, Llewellyn‐Thomas HA, Gladstone P, Cusimano R, O'rourke K, et al. Randomized, controlled trial of an interactive videodisc decision aid for patients with ischemic heart disease. Journal of General Internal Medicine. 2000;15(10):685-93.

9. Barry MJ, Cherkin DC, Chang Y, FJ JF, Skates S. A randomized trial of a multimedia shared decision-making program for men facing a treatment decision for benign prostatic hyperplasia. Disease Management and Clinical Outcomes. 1997;1(1):5-14.

10. Coylewright M, Dick S, Zmolek B, Askelin J, Hawkins E, Branda M, et al. PCI choice decision aid for stable coronary artery disease: A randomized trial. Circulation: Cardiovascular Quality and Outcomes. 2016;9(6):767-76.

11. McAlister FA, Man-Son-Hing M, Straus SE, Ghali WA, Anderson D, Majumdar SR, et al. Impact of a patient decision aid on care among patients with nonvalvular atrial fibrillation: A cluster randomized trial. Canadian Medical Association Journal. 2005;173(5):496-501.

12. Gagné ME, Légaré F, Moisan J, Boulet L-P. Impact of adding a decision aid to patient education in adults with asthma: A randomized clinical trial. PloS One. 2017;12(1):e0170055.

13. Slok AH, Kotz D, van Breukelen G, Chavannes NH, Rutten-van Mölken MP, Kerstjens HA, et al. Effectiveness of the Assessment of Burden of COPD (ABC) tool on health-related quality of life in patients with COPD: A cluster randomised controlled trial in primary and hospital care. BMJ Open. 2016;6(7):e011519.

14. Huang ES, Nathan AG, Cooper JM, Lee SM, Shin N, John PM, et al. Impact and feasibility of personalized decision support for older patients with diabetes: A pilot randomized trial. Medical Decision Making. 2017;37(5):611-7.

15. Weymiller AJ, Montori VM, Jones LA, Gafni A, Guyatt GH, Bryant SC, et al. Helping patients with type 2 diabetes mellitus make treatment decisions: Statin choice randomized trial. Archives of Internal Medicine. 2007;167(10):1076-82.

16. Nannenga MR, Montori VM, Weymiller AJ, Smith SA, Christianson TJ, Bryant SC, et al. A treatment decision aid may increase patient trust in the diabetes specialist. The Statin Choice randomized trial. Health Expectations. 2009;12(1):38-44.

17. Mann DM, Ponieman D, Montori VM, Arciniega J, McGinn T. The Statin Choice decision aid in primary care: A randomized trial. Patient Education and Counseling. 2010;80(1):138-40.

18. Perestelo-Pérez L, Rivero-Santana A, Boronat M, Sánchez-Afonso JA, Pérez-Ramos J, Montori VM, et al. Effect of the statin choice encounter decision aid in Spanish patients with type 2 diabetes: A randomized trial. Patient Education and Counseling. 2016;99(2):295-9.

19. Barry MJ, Fowler FJ, Mulley AG, Henderson JV, Wennberg JE. Patient reactions to a program designed to facilitate patient participation in treatment decisions for benign prostatic hyperplagia. Medical Care. 1995;33(8):771-82.

20. Mathers N, Ng CJ, Campbell MJ, Colwell B, Brown I, Bradley A. Clinical effectiveness of a patient decision aid to improve decision quality and glycaemic control in people with diabetes making treatment choices: A cluster randomised controlled trial (PANDAs) in general practice. BMJ Open. 2012;2(6):e001469.

21. Heisler M, Choi H, Palmisano G, Mase R, Richardson C, Fagerlin A, et al. Comparison of community health worker-led diabetes medication decision-making support for low-income Latino and African American adults with diabetes using e-health tools versus print materials: A randomized, controlled trial. Annals of Internal Medicine. 2014;161(10_Supplement):S13-S22.

22. Morisky DE, Green LW, Levine DM. Concurrent and predictive validity of a self-reported measure of medication adherence. Medical Care. 1986;24(1):67-74.

23. Bailey RA, Pfeifer M, Shillington AC, Harshaw Q, Funnell MM, VanWingen J, et al. Effect of a patient decision aid (PDA) for type 2 diabetes on knowledge, decisional self-efficacy, and decisional conflict. BMC Health Services Research. 2016;16(1):10.

24. Denig P, Schuling J, Haaijer-Ruskamp F, Voorham J. Effects of a patient oriented decision aid for prioritising treatment goals in diabetes: Pragmatic randomised controlled trial. BMJ: British Medical Journal. 2014;349:g5651.

25. Mullan RJ, Montori VM, Shah ND, Christianson TJ, Bryant SC, Guyatt GH, et al. The diabetes mellitus medication choice decision aid: A randomized trial. Archives of Internal Medicine. 2009;169(17):1560-8.

26. Karagiannis T, Liakos A, Branda ME, Athanasiadou E, Mainou M, Boura P, et al. Use of the Diabetes Medication Choice Decision Aid in patients with type 2 diabetes in Greece: A cluster randomised trial. BMJ Open. 2016;6(11):e012185.

27. Den Ouden H, Vos RC, Rutten GE. Effectiveness of shared goal setting and decision making to achieve treatment targets in type 2 diabetes patients: A cluster‐randomized trial (OPTIMAL). Health Expectations. 2017;20(5):1172-80.
