# Supplementary material for: Decision aids that facilitate elements of shared decision making in chronic illnesses: a systematic review
Source: Syst Rev. 2019 May 20;8:121. doi: 10.1186/s13643-019-1034-4 (PMC6528254; doi:10.1186/s13643-019-1034-4)
Supplement: Supplementary file 5 — DA effects. (DOCX 64 kb) [file 13643_2019_1034_MOESM5_ESM.docx]

**Additional file 5 DA effects**

Decision aid effects on SDM outcomes

**Decision aid effects on decisional conflict**

| **Study** | **Measurement moment** | **N intervention group included in analyses** | **N control group included in analyses** | **Measurement instrument** | **Standardized mean difference (95%-CI)**^a^ | **Risk of bias** |
| --- | --- | --- | --- | --- | --- | --- |
| Knops et al. 2014^1^ | 1 To 4 weeks post-encounter | 73 | 81 | Decisional Conflict Scale^b^ | -0.12 (-0.43 to 0.20) | High risk |
| Man-Son-Hing et al. 1999^2^ | 1 To 4 days post-encounter | 139 | 148 | Decisional Conflict Scale and two additional items to elicit patients' perceptions about the extent they were informed about 1) the benefits and risks of warfarin and 2) about benefits and risks of aspirin^b^ | -0.18 (-0.41 to 0.05) | Unclear risk |
| Thomas et al. 2013^3^ | 1 Week post-encounter | 39 | 17 | A modified version of the Decisional Conflict Scale^b^ | 0.29 (-0.29 to 0.86) | Unclear risk |
| Korteland et al. 2017^4^ | Immediately post-encounter | 66 | 70 | Decisional Conflict Scale (score range: 0-100)^b,c^ | SMD could not be calculated, but there was a non-significant difference in DCS score (median intervention group = 24 (0-69); median control group = 24 (0-72)) | Unclear risk |
| Thomson et al. 2007^5^ | Immediately post-encounter | 53 | 55 | Decisional Conflict Scale^b^ | -0.40 (-0.78 to -0.02) | High risk |
| Coylewright et al. 2016^6^ | Immediately post-encounter | 58 | 48 | Decisional Conflict Scale^b^ | -0.20 (-0.59 to 0.18) | High risk |
| McAlister et al. 2005^7^ | 2 Weeks post-intervention | 219 | 215 | Decisional Conflict Scale^b^ | -0.20 (-0.39 to -0.01) | High risk |
| Huang et al. 2017^8^ | Immediately post-encounter | 75 | 25 | The low literacy version of the Decisional Conflict Scale^b^ | -0.42 (-0.88 to 0.04) | High risk |
| Nannenga et al. 2009^9d^ | Immediately post-encounter | 51 | 46 | Decisional Conflict Scale^b^ | -0.88 (-1.30 to -0.46) | High risk |
| Mann et al. 2010^10^ | Immediately post-encounter | 80 | 70 | Decisional Conflict Scale^b^ | -0.25 (-0.58 to 0.07)^e^ | Unclear risk |
| Weymiller et al. 2007^11d^ | Immediately post-encounter | 51 | 46 | Decisional Conflict Scale^b^ | -0.89 (-1.31 to -0.47) | High risk |
| Perestelo-Perez et al. 2016^12^ | Immediately post-encounter | 78 | 77 | Decisional Conflict Scale^b^ | 0.00 (-0.31 to 0.32) | High risk |
| Mathers et al. 2012^13^ | Immediately post-encounter | 89 | 78 | Decisional Conflict Scale^b^ | -0.49 (-0.80 to -0.18) | High risk |
| Heisler et al. 2014^14^ | Immediately post-encounter | 91 | 95 | Decisional Conflict Scale^b^ | -0.01 (-0.29 to 0.28) | Unclear risk |
| Bailey et al. 2016^15^ | 4 To 6 weeks post-intervention | 114 | 111 | Decisional Conflict Scale^b^ | -0.78 (-1.05 to -0.51) | Unclear risk |
| Mullan et al. 2009^16^ | Immediately post-encounter | 48 | 37 | Decisional Conflict Scale^b^ | -0.08 (-0.51 to 0.34) | High risk |
| Karagiannis et al. 2016^17^ | Immediately post-encounter | 101 | 103 | 13-Item modified version of the Decisional Conflict Scale^b^ | -0.13 (-0.40 to 0.15) | High risk |
| Gagné et al. 2017^18^ | 2 Months post-intervention | 26 | 25 | French version of the Decisional Conflict Scale^b^ | -0.15 (-0.70 to 0.40) | Low risk |

^a^Control group is reference group ^b^Higher scores indicate less favorable decisional conflict ^c^Scores range from 0 to 100 ^d^Weymiller et al. 2007 and Nannenga et al. 2009 are based on the same RCT
^e^SD of Weymiller et al. 2007 is imputed to calculate the SMD and its 95%-CI

**Decision aid effects on treatment satisfaction**

| **Study** | **Measurement moment** | **N intervention group included in analyses** | **N control group included in analyses** | **Measurement instrument** | **Standardized mean difference (95%-CI)**^a^ | **Risk of bias** |
| --- | --- | --- | --- | --- | --- | --- |
| Denig et al. 2014^19^ | 3 To 4 months post-encounter | 205 | 108 | Patients' Evaluation of Quality of Diabetes care (PEQD) questionnaire^b^ | -0.05 (-0.28 to 0.19) | High risk |
| Slok et al. 2016^20^ | 12 Months post-encounter | 141 | 152 | Patient Assessment of Chronic Illness Care (PACIC)^b^ | 0.26 (0.03 to 0.49) | High risk |

^a^Control group is reference group ^b^Higher scores indicate more favorable perceived quality of care

**Decision aid effects on decision self-efficacy**

| **Study** | **Measurement moment** | **N intervention group included in analyses** | **N control group included in analyses** | **Measurement instrument** | **Standardized mean difference (95%-CI)**^a^ | **Risk of bias** |
| --- | --- | --- | --- | --- | --- | --- |
| Bailey et al. 2016^15^ | 4 To 6 weeks post-intervention | 114 | 111 | Decision Self Efficacy Scale (DSES)^b^ | 0.38 (0.12 to 0.65) | Unclear risk |

^a^Control group is reference group ^b^Higher scores indicate more favorable decision self-efficacy

**Decision aid effects on trust in physician**

| **Study** | **Measurement moment** | **N intervention group included in analyses** | **N control group included in analyses** | **Measurement instrument** | **Standardized mean difference (95%-CI)**^a^ | **Risk of bias** |
| --- | --- | --- | --- | --- | --- | --- |
| Nannenga et al. 2009^9^ | Immediately post-encounter | 51 | 46 | Trust in Physician Scale^b^ | 0.29 (-0.11 to 0.69) | High risk |
| Mullan et al. 2009^16^ | Immediately post-encounter | 48 | 37 | 9-Item version of the Trust in Physician Scale^b^ | 0.23 (-0.20 to 0.66) | High risk |

^a^Control group is reference group ^b^Higher scores indicate more favorable trust in physician

**Decision aid effects on patient participation in decision making**

| **Study** | **Measurement moment** | **N intervention group included in analyses** | **N control group included in analyses** | **Measurement instrument** | **Standardized mean difference (95%-CI)**^a^ | **Odds ratio (95%-CI)** | **Risk of bias** |
| --- | --- | --- | --- | --- | --- | --- | --- |
| Man-Son-Hing et al. 1999^2^ | 1 To 4 days post-encounter | 139 | 148 | 5-Point Likert scale to judge the relative strength of the patient's personal input into the choice versus their physician | Not applicable | 1.23 (0.77 to 1.97)^b^ | Unclear risk |
| Coylewright et al. 2016^6^ | During the encounter (analyzes based on recorded encounters) | 34 | 20 | Observing Patient Involvement in Decision Making Scale (OPTION12)^c^ | 0.50 (-0.06 to 1.06) | Not applicable | High risk |
| Nannenga et al. 2009^9^ | During the encounter (analyzes based on recorded encounters) | 52 | 46 | Observing Patient Involvement in Decision Making Scale (OPTION12)^c^ | 1.08 (0.66 to 1.51) | Not applicable | High risk |
| Mullan et al. 2009^16^ | During the encounter | 30 | 21 | Observing Patient Involvement in Decision Making Scale (OPTION12)^c^ | 1.36 (0.74 to 1.98) | Not applicable | High risk |

^a^Control group is reference group ^b^Event is regarded as patient reported that he/she made the decision, rather than his/her physician; control group is reference group
^c^Higher scores indicate more patient involvement in decision making

**Decision aid effects on conversation satisfaction**

| **Study** | **Measurement moment** | **N intervention group included in analyses** | **N control group included in analyses** | **Measurement instrument** | **Standardized mean difference (95%-CI)**^a^ | **Risk of bias** |
| --- | --- | --- | --- | --- | --- | --- |
| Knops et al. 2014^1^ | 1 To 4 weeks post-encounter | 74 | 80 | Patient Satisfaction Questionnaire^b^ | 0.06 (-0.26 to 0.37) | High risk |

^a^Control group is reference group ^b^Higher scores indicate more favorable conversation satisfaction

**Decision aid effects on satisfaction with the decision making process**

| **Study** | **Measurement moment** | **N intervention group included in analyses** | **N control group included in analyses** | **Measurement instrument** | **Standardized mean difference (95%-CI)**^a^ | **Risk of bias** |
| --- | --- | --- | --- | --- | --- | --- |
| Man-Son-Hing et al. 1999^2^ | 1 To 4 days post-encounter | 146 | 138 | 6 Items using a 5-point Likert scale | -0.07 (-0.30 to 0.16) | Unclear risk |
| Morgan et al. 2000^21^ | At time of treatment decision | 90 | 97 | A modified version of the 12-item decision making process questionnaire developed by Barry et al. (1997)^22b^ | 0.07 (-0.22 to 0.36) | High risk |
| Perestelo-Perez et al. 2016^12^ | Immediately post-encounter | 80 | 73 | A modified version of the 12-item decision making process questionnaire developed by Barry et al. (1995)^23b^ | 0.42 (0.10 to 0.74) | High risk |

^a^Control group is reference group ^b^Higher scores indicate more favorable satisfaction with the decision-making process

**Decision aid effects on treatment decision (preference)**

| **Study** | **Measurement moment** | **N intervention group included in analyses** | **N control group included in analyses** | **Measurement instrument** | **Odds ratio (95%-CI)** | **Risk of bias** |
| --- | --- | --- | --- | --- | --- | --- |
| Knops et al. 2014^1^ | 9 To 10 months post-encounter | 91 | 87 | Extracted from the medical record | 0.94 (0.52 to 1.71)^a^ | Unclear risk |
| Man-Son-Hing et al. 1999^2^ | 1 To 4 days post-encounter | 139 | 148 | Questionnaire in which participants were, among others, asked what decision regarding antithrombotic therapy was made | 0.73 (0.33 to 1.59)^b^ | Unclear risk |
| Thomas et al. 2013^3^ | 3 Months post-encounter | 39 | 20 | Unclear | 0.59 (0.14 to 2.47)^c^ | Unclear risk |
| Thomson et al. 2007^5^ | 3 Months post-encounter | 53 | 56 | Extracted from the primary care record | 0.33 (0.12 to 0.95)^d^ | High risk |
| Morgan et al. 2000^21^ | Unclear | 90 | 97 | Recorded | 0.45 (0.24 to 0.84)^e^ | High risk |
| Huang et al. 2017^8^ | Immediately post-encounter | 75 | 25 | Physician survey (a change in goal was defined as a 0,5% increase or decrease in HbA1c goal from pre-survey to post-survey responses) | 0.40 (0.15 to 1.07)^f^ | High risk |
| Weymiller et al. 2007^11^ | Immediately post-encounter | 23 | 19 | Percentage of participants not receiving statin therapy at baseline deciding to start statin therapy (unclear whether this is measured based on recordings or by questionnaires) | 0.61 (0.15 to 2.51)^g^ | High risk |
| Mullan et al. 2009^16^ | Immediately post-encounter | 48 | 37 | Physician survey | 0.55 (0.21 to 1.48)^h^ | High risk |

^a^Event is regarded as not choosing elective aneurysm impair; control group is reference group
^b^Event is regarded as deciding to take warfarin; control group is reference group
^c^Event is regarded as ICD implantation within 3 months; control group is reference group
^d^Event is regarded as starting or continuing warfarin; control group is reference group
^e^Event is regarded as revascularization as initial decision; control group is reference group
^f^Event is regarded as goal stayed the same; control group is reference group
^g^Event is regarded as deciding not to take statin therapy; control group is reference group
^h^Event is regarded as continue taking current medications; control group is reference group

**Decision aid effects on proportion undecided**

| **Study** | **Measurement moment** | **N intervention group included in analyses** | **N control group included in analyses** | **Measurement instrument** | **Odds ratio (95%-CI)** | **Risk of bias** |
| --- | --- | --- | --- | --- | --- | --- |
| Man-Son-Hing et al. 1999^2^ | 1 To 4 days post-encounter | 139 | 148 | Participants were asked to indicate whether a decision regarding the choice of antithrombotic therapy had been made in conjunction with their physician | 0.11 (0.01 to 0.90)^a^ | Unclear risk |
| Mathers et al. 2012^13^ | Immediately post-encounter | 89 | 78 | Participants were asked a question on readiness for decision making | 0.76 (0.28 to 2.07)^b^ | High risk |

^a^Event was regarded as not being able to make a definite choice; control group is reference group
^b^Event is regarded as being undecided; control group is reference group

**Decision aid effects on conversation duration**

| **Study** | **Measurement moment** | **N intervention group included in analyses** | **N control group included in analyses** | **Measurement instrument** | **Standardized mean difference (95%-CI)**^a^ | **Risk of bias** |
| --- | --- | --- | --- | --- | --- | --- |
| Nannenga et al. 2009^9^ | During the encounter | 52 | 46 | Videotapes of the encounters | 0.22 (-0.17 to 0.62) | High risk |
| Perestelo-Perez et al. 2016^12^ | During the encounter | 61 | 63 | Documented by the physician | -0.18 (-0.53 to 0.17) | High risk |
| Mathers et al. 2012^13^ | During the encounter | 89 | 78 | Timed by the researcher from the point the patient entered the consultation room to the time patient left | -0.19 (-0.50 to 0.11) | High risk |

^a^Control group is reference group

**Decision aid effects on knowledge**

| **Study** | **Measurement moment** | **N intervention group included in analyses** | **N control group included in analyses** | **Measurement instrument** | **Standardized mean difference (95%-CI)**^a^ | **Risk of bias** |
| --- | --- | --- | --- | --- | --- | --- |
| Knops et al. 2014^1^ | 1 To 4 weeks post-encounter | 80 | 84 | 13 Items of the Dutch multiple-choice Aneurysm Knowledge Questionnaire^b^ | 0.28 (-0.03 to 0.59) | Unclear risk |
| Thomas et al. 2013^3^ | Immediately post-encounter | 39 | 20 | A developed 13-item questionnaire to assess participant's knowledge of SCA, associated risk factors, and ICD therapy^b^ | 0.45 (-0.09 to 1.00) | Unclear risk |
| El-Jawahri et al. 2016^24^ | Immediately post-encounter | 123 | 123 | 5 True/false items and 1 multiple choice item^b^ | 0.76 (0.50 to 1.01) | High risk |
| Thomson et al. 2007^5^ | Immediately post-encounter | 53 | 55 | 23 True/false items about atrial fibrillation and stroke^b^ | 0.04 (-0.34 to 0.42) | High risk |
| Morgan et al. 2000^21^ | Time of treatment decision | 90 | 97 | 20 True/false items to assess knowledge deemed necessary for an informed treatment decision. This item set was reduced to 15 for patients who were not eligible for angioplasty^b^ | 0.74 (0.45 to 1.04) | High risk |
| Nannenga et al. 2009^9^ | Immediately post-encounter | 51 | 46 | 16 Knowledge items^b^ | 0.70 (0.29 to 1.11) | High risk |
| Heisler et al. 2014^14^ | Immediately post-encounter | 92 | 95 | Items regarding knowledge about anti-hyperglycemic medications | -0.06 (-0.35 to 0.23) | Unclear risk |
| Bailey et al. 2016^15^ | 4 To 6 weeks post-intervention | 114 | 111 | A developed questionnaire to assess understanding of how different treatments differ in terms of their impact on glycemic control (amount and durability), impact on weight, risk of hypoglycemia and other adverse events, route of administration, frequency of dose administration and blood glucose monitoring, and financial costs^b^ | 1.09 (0.81 to 1.37) | Unclear risk |
| Karagiannis et al. 2016^17^ | Immediately post-encounter | 101 | 103 | 6-Item questionnaire addressing general knowledge about T2DM management and medications^b^ | -0.03 (-0.31 to 0.24) | High risk |
| Gagné et al. 2017^18^ | 2 Months post-intervention | 26 | 25 | Questionnaire de connaissances sur l'asthme de langue francaise (QCALF)^b^ | 0.32 (-0.23 to 0.87) | Low risk |

^a^Control group is reference group ^b^Higher scores indicate greater knowledge

Decision aid effects on patient-reported outcomes

**Decision aid effects on diabetes care self-efficacy**

| **Study** | **Measurement moment** | **N intervention group included in analyses** | **N control group included in analyses** | **Measurement instrument** | **Standardized mean difference (95%-CI)**^a^ | **Risk of bias** |
| --- | --- | --- | --- | --- | --- | --- |
| Heisler et al. 2014^14^ | Immediately post-encounter | 92 | 94 | Unclear | 0.27 (-0.02 to 0.56) | Unclear risk |

^a^Control group is reference group

**Decision aid effects on (health-related) quality of life**

| **Study** | **Measurement moment** | **N intervention group included in analyses** | **N control group included in analyses** | **Measurement instrument** | **Standardized mean difference (95%-CI)**^a^ | **Risk of bias** |
| --- | --- | --- | --- | --- | --- | --- |
| Knops et al. 2014^1^ | 1 To 4 weeks post-encounter | 80 | 84 | 12-Item Short Form Health Survey (SF-12)^b^ | 0.10 (-0.21 to 0.41) | Unclear risk |
| Denig et al. 2014^19^ | 3 To 4 months post-encounter | 203 | 105 | Dutch version of the EuroQol (EQ-5D)^b^ | -0.05 (-0.28 to 0.19) | High risk |
| Slok et al. 2016^20^ | 18 Months post-encounter | 144 | 152 | COPD Assessment Test (CAT)^b^ | 0.05 (-0.18 to 0.27) | High risk |

^a^Control group is reference group ^b^Higher scores indicate more favorable (health-related) quality of life

**Decision aid effects on health status**

| **Study** | **Measurement moment** | **N intervention group included in analyses** | **N control group included in analyses** | **Measurement instrument** | **Standardized mean difference (95%-CI)**^a^ | **Risk of bias** |
| --- | --- | --- | --- | --- | --- | --- |
| Mullan et al. 2009^16^ | 6 Months post-encounter | 47 | 37 | Asking patients by telephone to rate their health as excellent, very good, good, fair, or poor^b^ | -0.03 (-0.46 to 0.40) | High risk |
| Slok et al. 2016^20^ | 6 Months post-encounter | 160 | 161 | St. George's Respiratory Questionnaire (SGRQ)^c^ | -0.10 (-0.32 to 0.12) | High risk |

^a^Control group is reference group ^b^Higher scores indicate more favorable self-reported health ^c^Higher scores indicate more favorable disease-specific health status

**Decision aid effects on illness distress**

| **Study** | **Measurement moment** | **N intervention group included in analyses** | **N control group included in analyses** | **Measurement instrument** | **Standardized mean difference (95%-CI)**^a^ | **Risk of bias** |
| --- | --- | --- | --- | --- | --- | --- |
| Perestelo-Perez et al. 2016^12^ | 3 Months post-encounter | 67 | 64 | Problem Areas In Diabetes (PAID)^b^ | 0.17 (-0.17 to 0.51) | High risk |
| Heisler et al. 2014^14^ | 3 Months post-encounter | 87 | 89 | Diabetes Distress Scale^b^ | -0.39 (-0.68 to -0.09) | Unclear risk |
| Denig et al. 2014^19^ | 3 To 4 months post-encounter | 204 | 107 | Problem Areas In Diabetes (PAID)^b^ | 0.23 (0.00 to 0.47) | High risk |

^a^Control group is reference group ^b^Higher scores indicate less favorable diabetes distress

**Decision aid effects on anxiety**

| **Study** | **Measurement moment** | **N intervention group included in analyses** | **N control group included in analyses** | **Measurement instrument** | **Standardized mean difference (95%-CI)**^a^ | **Risk of bias** |
| --- | --- | --- | --- | --- | --- | --- |
| Knops et al. 2014^1^ | 1 To 4 weeks post-encounter | 81 | 85 | Hospital Anxiety and Depression Scale (HADS)^b^ | -0.16 (-0.46 to 0.15) | Unclear risk |
| Fraenkel et al. 2012^25^ | Immediately post-encounter | 69 | 66 | Spielberger State Anxiety Index^b^ | -0.12 (-0.46 to 0.22) | Unclear risk |
| Korteland et al. 2017^4^ | Immediately post-encounter | 67 | 71 | The Hospital Anxiety and Depression Scale (HADS)^b,c^ | SMD could not be calculated, but there was a significant difference in HADS score (median intervention group = 6 (0-33); median control group = 9 (0-41)) | Unclear risk |
| Thomson et al. 2007^5^ | Immediately post-encounter | 53 | 55 | State Trait Anxiety Inventory (STAI)^b^ | -0.11 (-0.49 to 0.28) | High risk |
| Perestelo-Perez et al. 2016^12^ | Immediately post-encounter | 80 | 77 | Spanish version of the State Trait Anxiety Inventory (STAI)^b^ | -0.09 (-0.40 to 0.23) | High risk |

^a^Control group is reference group ^b^Higher scores indicate more anxiety
^c^Scores range from 0 to 42

Decision aid effects on surrogate outcomes

**Decision aid effects on total cholesterol**

| **Study** | **Measurement moment** | **N intervention group included in analyses** | **N control group included in analyses** | **Measurement instrument** | **Standardized mean difference (95%-CI)**^a^ | **Risk of bias** |
| --- | --- | --- | --- | --- | --- | --- |
| den Ouden et al. 2017^26^ | 24 Months post-encounter | 66 | 75 | Total cholesterol in mmol/L (measured by standard enzymatic techniques (Cobas 8000 machine)) | 0.15 (-0.18 to 0.49) | High risk |

^a^Control group is reference group

**Decision aid effects on blood pressure**

| **Study** | **Measurement moment** | **N intervention group included in analyses** | **N control group included in analyses** | **Measurement instrument** | **Standardized mean difference (95%-CI)**^a^ | **Risk of bias** |
| --- | --- | --- | --- | --- | --- | --- |
| den Ouden et al. 2017^26^ | 24 Months post-encounter | 66 | 73 | Systolic blood pressure in mm Hg (measured by two measurements after at least 10 minutes rest while participants were seated with the cuff on the predominant arm at the level of the heart) | -0.27 (-0.60 to 0.07) | High risk |

^a^Control group is reference group

**Decision aid effects on glycemic control**

| **Study** | **Measurement moment** | **N intervention group included in analyses** | **N control group included in analyses** | **Measurement instrument** | **Standardized mean difference (95%-CI)**^a^ | **Risk of bias** |
| --- | --- | --- | --- | --- | --- | --- |
| Mathers et al. 2012^13^ | 6 Months post-encounter | 89 | 78 | Reported by the healthcare provider based on latest HbA1c on the medical records | 0.24 (-0.06 to 0.55) | High risk |
| Heisler et al. 2014^14^ | 3 Months post-encounter | 86 | 89 | HbA1c in % (measured by the Bayer DCA 2000+ point-of-care analyzer) | -0.06 (-0.35 to 0.24) | Unclear risk |
| Mullan et al. 2009^16^ | 6 Months post-encounter | 48 | 37 | HbA1c in % (extracted from the medical record) | -0.01 (-0.44 to 0.42) | High risk |
| Karagiannis et al. 2016^17^ | 3 Months post-encounter | 91 | 96 | HbA1c in % (measured at a local lab or lab of patient’s choice) | 0.28 (0.00 to 0.57) | High risk |
| den Ouden et al. 2017^26^ | 24 Months post-encounter | 65 | 72 | HbA1c in mmol/mol (measured by a high-performance liquid chromatography (Tosoh G8 machine)) | 0.22 (-0.12 to 0.55) | High risk |

^a^Control group is reference group

Decision aid effects on clinical outcomes

**Decision aid effects on Body Mass Index**

| **Study** | **Measurement moment** | **N intervention group included in analyses** | **N control group included in analyses** | **Measurement instrument** | **Standardized mean difference (95%-CI)**^a^ | **Risk of bias** |
| --- | --- | --- | --- | --- | --- | --- |
| Karagiannis et al. 2016^17^ | 6 Months post-encounter | 84 | 83 | BMI (measured by physician) | -0.20 (-0.50 to 0.11) | High risk |
| den Ouden et al. 2017^26^ | 24 Months post-encounter | 62 | 60 | BMI (measured by general practitioner) | -0.14 (-0.50 to 0.21) | High risk |

^a^Control group is reference group

**Decision aid effects on smoking status**

| **Study** | **Measurement moment** | **N intervention group included in analyses** | **N control group included in analyses** | **Measurement instrument** | **Odds ratio (95%-CI)** | **Risk of bias** |
| --- | --- | --- | --- | --- | --- | --- |
| Denig et al. 2014^19^ | 6 Months post-encounter | 184 | 98 | Extracted from the medical record | 0.40 (0.04 to 3.77)^a^ | High risk |

^a^Event is regarded as smoking; control group is reference group

**Decision aid effects on adherence**

| **Study** | **Measurement moment** | **N intervention group included in analyses** | **N control group included in analyses** | **Measurement instrument** | **Standardized mean difference (95%-CI)**^a^ | **Odds ratio (95%-CI)** | **Risk of bias** |
| --- | --- | --- | --- | --- | --- | --- | --- |
| Man-Son-Hing et al. 1999^2^ | 6 Months post-encounter | 129 | 134 | Participants were asked by telephone which therapy they were currently taking | Not applicable | 1.48 (0.51 to 4.27)^b^ | Unclear risk |
| Weymiller et al. 2007^11^ | 3 Months post-encounter | 33 | 29 | Mailed survey (and telephone calls for non-responders) to determine whether participants had missed any doses in the last week | Not applicable | 1.9 (0.40 to 9.80)^c^ | High risk |
| Heisler et al. 2014^14^ | 3 Months post-encounter | 87 | 89 | A self-reported measure of medication adherence developed by Morisky et al (1986)^27d^ | 0.07 (-0.23 to 0.37) | Not applicable | Unclear risk |
| Gagné et al. 2017^18^ | 2 Months post-intervention | 26 | 25 | A 4-item face-to-face interviewer-administered questionnaire | Not applicable | 2.05 (0.67 to 6.24)^e^ | Low risk |

^a^Control group is reference group ^b^Event is regarded as continuing to take the therapy that was initially chosen; control group is reference group
^c^Event is regarded as not missing any dose in the last week; control group is reference group ^d^Higher scores indicate more favorable medication adherence ^e^Event is regarded as appropriate use of pharmacotherapy (asthma drugs). For participants to be considered as appropriate users of asthma drugs, they needed to meet eleven hierarchical criteria, which included using their controller medications for the same number of times every day and at an adequate frequency; control group is reference group

**Decision aid effects on achieving treatment goals**

| **Study** | **Measurement moment** | **N intervention group included in analyses** | **N control group included in analyses** | **Measurement instrument** | **Standardized mean difference (95%-CI)**^a^ | **Risk of bias** |
| --- | --- | --- | --- | --- | --- | --- |
| Gagné et al. 2017^18^ | 2 Months post-intervention | 26 | 25 | The clinical and physiological subscales of the Asthma Control Scoring System (ACSS)^b^ | -0.05 (-0.60 to 0.50) | Low risk |

^a^Control group is reference group ^b^Higher scores indicate better asthma control

**References**

1. Knops A, Goossens A, Ubbink D, Balm R, Koelemay M, Vahl A, et al. A decision aid regarding treatment options for patients with an asymptomatic abdominal aortic aneurysm: A randomised clinical trial. European Journal of Vascular and Endovascular Surgery. 2014;48(3):276-83.
2. Man-Son-Hing M, Laupacis A, O'Connor AM, Biggs J, Drake E, Yetisir E, et al. A patient decision aid regarding antithrombotic therapy for stroke prevention in atrial fibrillation: A randomized controlled trial. JAMA: Journal of the American Medical Association. 1999;282(8):737-43.
3. Thomas KL, Zimmer LO, Dai D, Al-Khatib SM, LaPointe NMA, Peterson ED. Educational videos to reduce racial disparities in ICD therapy via innovative designs (VIVID): A randomized clinical trial. American Heart Journal. 2013;166(1):157-63.
4. Korteland NM, Ahmed Y, Koolbergen DR, Brouwer M, de Heer F, Kluin J, et al. Does the use of a decision aid improve decision making in prosthetic heart valve selection?: A multicenter randomized trial. Circulation: Cardiovascular Quality and Outcomes. 2017;10(2):e003178.
5. Thomson RG, Eccles MP, Steen IN, Greenaway J, Stobbart L, Murtagh MJ, et al. A patient decision aid to support shared decision-making on anti-thrombotic treatment of patients with atrial fibrillation: Randomised controlled trial. BMJ Quality & Safety. 2007;16(3):216-23.
6. Coylewright M, Dick S, Zmolek B, Askelin J, Hawkins E, Branda M, et al. PCI choice decision aid for stable coronary artery disease: A randomized trial. Circulation: Cardiovascular Quality and Outcomes. 2016;9(6):767-76.
7. McAlister FA, Man-Son-Hing M, Straus SE, Ghali WA, Anderson D, Majumdar SR, et al. Impact of a patient decision aid on care among patients with nonvalvular atrial fibrillation: A cluster randomized trial. Canadian Medical Association Journal. 2005;173(5):496-501.
8. Huang ES, Nathan AG, Cooper JM, Lee SM, Shin N, John PM, et al. Impact and feasibility of personalized decision support for older patients with diabetes: A pilot randomized trial. Medical Decision Making. 2017;37(5):611-7.
9. Nannenga MR, Montori VM, Weymiller AJ, Smith SA, Christianson TJ, Bryant SC, et al. A treatment decision aid may increase patient trust in the diabetes specialist. The Statin Choice randomized trial. Health Expectations. 2009;12(1):38-44.
10. Mann DM, Ponieman D, Montori VM, Arciniega J, McGinn T. The Statin Choice decision aid in primary care: A randomized trial. Patient Education and Counseling. 2010;80(1):138-40.
11. Weymiller AJ, Montori VM, Jones LA, Gafni A, Guyatt GH, Bryant SC, et al. Helping patients with type 2 diabetes mellitus make treatment decisions: Statin choice randomized trial. Archives of Internal Medicine. 2007;167(10):1076-82.
12. Perestelo-Pérez L, Rivero-Santana A, Boronat M, Sánchez-Afonso JA, Pérez-Ramos J, Montori VM, et al. Effect of the statin choice encounter decision aid in Spanish patients with type 2 diabetes: A randomized trial. Patient Education and Counseling. 2016;99(2):295-9.
13. Mathers N, Ng CJ, Campbell MJ, Colwell B, Brown I, Bradley A. Clinical effectiveness of a patient decision aid to improve decision quality and glycaemic control in people with diabetes making treatment choices: A cluster randomised controlled trial (PANDAs) in general practice. BMJ Open. 2012;2(6):e001469.
14. Heisler M, Choi H, Palmisano G, Mase R, Richardson C, Fagerlin A, et al. Comparison of community health worker-led diabetes medication decision-making support for low-income Latino and African American adults with diabetes using e-health tools versus print materials: A randomized, controlled trial. Annals of Internal Medicine. 2014;161(10_Supplement):S13-S22.
15. Bailey RA, Pfeifer M, Shillington AC, Harshaw Q, Funnell MM, VanWingen J, et al. Effect of a patient decision aid (PDA) for type 2 diabetes on knowledge, decisional self-efficacy, and decisional conflict. BMC Health Services Research. 2016;16(1):10.
16. Mullan RJ, Montori VM, Shah ND, Christianson TJ, Bryant SC, Guyatt GH, et al. The diabetes mellitus medication choice decision aid: A randomized trial. Archives of Internal Medicine. 2009;169(17):1560-8.
17. Karagiannis T, Liakos A, Branda ME, Athanasiadou E, Mainou M, Boura P, et al. Use of the Diabetes Medication Choice Decision Aid in patients with type 2 diabetes in Greece: A cluster randomised trial. BMJ Open. 2016;6(11):e012185.
18. Gagné ME, Légaré F, Moisan J, Boulet L-P. Impact of adding a decision aid to patient education in adults with asthma: A randomized clinical trial. PloS One. 2017;12(1):e0170055.
19. Denig P, Schuling J, Haaijer-Ruskamp F, Voorham J. Effects of a patient oriented decision aid for prioritising treatment goals in diabetes: Pragmatic randomised controlled trial. BMJ: British Medical Journal. 2014;349:g5651.
20. Slok AH, Kotz D, van Breukelen G, Chavannes NH, Rutten-van Mölken MP, Kerstjens HA, et al. Effectiveness of the Assessment of Burden of COPD (ABC) tool on health-related quality of life in patients with COPD: A cluster randomised controlled trial in primary and hospital care. BMJ Open. 2016;6(7):e011519.
21. Morgan MW, Deber RB, Llewellyn‐Thomas HA, Gladstone P, Cusimano R, O'rourke K, et al. Randomized, controlled trial of an interactive videodisc decision aid for patients with ischemic heart disease. Journal of General Internal Medicine. 2000;15(10):685-93.
22. Barry MJ, Cherkin DC, Chang Y, FJ JF, Skates S. A randomized trial of a multimedia shared decision-making program for men facing a treatment decision for benign prostatic hyperplasia. Disease Management and Clinical Outcomes. 1997;1(1):5-14.
23. Barry MJ, Fowler FJ, Mulley AG, Henderson JV, Wennberg JE. Patient reactions to a program designed to facilitate patient participation in treatment decisions for benign prostatic hyperplagia. Medical Care. 1995;33(8):771-82.
24. El-Jawahri A, Paasche-Orlow MK, Matlock D, Stevenson LW, Lewis EF, Stewart G, et al. Randomized, controlled trial of an advance care planning video decision support tool for patients with advanced heart failure. Circulation. 2016;134(1):52-60.
25. Fraenkel L, Street RL, Towle V, O'leary JR, Iannone L, Ness PH, et al. A pilot randomized controlled trial of a decision support tool to improve the quality of communication and decision‐making in individuals with atrial fibrillation. Journal of the American Geriatrics Society. 2012;60(8):1434-41.
26. Den Ouden H, Vos RC, Rutten GE. Effectiveness of shared goal setting and decision making to achieve treatment targets in type 2 diabetes patients: A cluster‐randomized trial (OPTIMAL). Health Expectations. 2017;20(5):1172-80.
27. Morisky DE, Green LW, Levine DM. Concurrent and predictive validity of a self-reported measure of medication adherence. Medical Care. 1986;24(1):67-74.
